# Discovery and Mechanistic Characterization of Selective Inhibitors of H_2_S-producing Enzyme: 3-Mercaptopyruvate Sulfurtransferase (3MST) Targeting Active-site Cysteine Persulfide

**DOI:** 10.1038/srep40227

**Published:** 2017-01-12

**Authors:** Kenjiro Hanaoka, Kiyoshi Sasakura, Yusuke Suwanai, Sachiko Toma-Fukai, Kazuhito Shimamoto, Yoko Takano, Norihiro Shibuya, Takuya Terai, Toru Komatsu, Tasuku Ueno, Yuki Ogasawara, Yukihiro Tsuchiya, Yasuo Watanabe, Hideo Kimura, Chao Wang, Masanobu Uchiyama, Hirotatsu Kojima, Takayoshi Okabe, Yasuteru Urano, Toshiyuki Shimizu, Tetsuo Nagano

**Affiliations:** 1 Graduate School of Pharmaceutical Sciences, The University of Tokyo, 7-3-1 Hongo, Bunkyo-ku, Tokyo 113-0033, Japan; 2 Department of Molecular Pharmacology, National Institute of Neuroscience, National Center of Neurology and Psychiatry, 4-1-1 Ogawa-Higashi, Kodaira, Tokyo 187-8502, Japan; 3 PRESTO (Japan) Science and Technology Agency (JST), 4-1-8 Honcho Kawaguchi, Saitama 332-0012, Japan; 4 Department of Analytical Biochemistry, Meiji Pharmaceutical University, 2-522-1 Noshio, Kiyose, Tokyo 204-8588, Japan; 5 High Technology Research Center, Pharmacology, Showa Pharmaceutical University, Machidashi 194-8543, Tokyo, Japan; 6 Advanced Elements Chemistry Research Team, RIKEN Center for Sustainable Resource Science, and Elements Chemistry Laboratory, RIKEN, 2-1 Hirosawa, Wako-shi, Saitama 351-0198, Japan; 7 Drug Discovery Initiative, The University of Tokyo, 7-3-1 Hongo, Bunkyo-ku, Tokyo 113-0033, Japan; 8 Graduate School of Medicine, The University of Tokyo, 7-3-1 Hongo, Bunkyo-ku, Tokyo 113-0033, Japan; 9 CREST (Japan) Agency for Medical Research and Development (AMED), 1-7-1 Otemachi, Chiyoda-ku, Tokyo 100-0004, Japan

## Abstract

Very recent studies indicate that sulfur atoms with oxidation state 0 or −1, called sulfane sulfurs, are the actual mediators of some physiological processes previously considered to be regulated by hydrogen sulfide (H_2_S). 3-Mercaptopyruvate sulfurtransferase (3MST), one of three H_2_S-producing enzymes, was also recently shown to produce sulfane sulfur (H_2_S_n_). Here, we report the discovery of several potent 3MST inhibitors by means of high-throughput screening (HTS) of a large chemical library (174,118 compounds) with our H_2_S-selective fluorescent probe, HSip-1. Most of the identified inhibitors had similar aromatic ring-carbonyl-*S*-pyrimidone structures. Among them, compound **3** showed very high selectivity for 3MST over other H_2_S/sulfane sulfur-producing enzymes and rhodanese. The X-ray crystal structures of 3MST complexes with two of the inhibitors revealed that their target is a persulfurated cysteine residue located in the active site of 3MST. Precise theoretical calculations indicated the presence of a strong long-range electrostatic interaction between the persulfur anion of the persulfurated cysteine residue and the positively charged carbonyl carbon of the pyrimidone moiety of the inhibitor. Our results also provide the experimental support for the idea that the 3MST-catalyzed reaction with 3-mercaptopyruvate proceeds via a ping-pong mechanism.

Hydrogen sulfide (H_2_S) plays roles in many physiological processes in mammals, including relaxation of vascular smooth muscles^1^, regulation of inflammation^2^, and inhibition of insulin signaling^3^. H_2_S generated by many prokaryotic species is also related to antibiotic resistance, serving to mitigate oxidative stress imposed by antibiotics^4^. Thus, H_2_S is an important reactive sulfur species not only in mammals, but also in bacteria. More recently, it has been proposed that sulfane sulfurs^5^, i.e., sulfur atoms with oxidation state 0 or −1, existing in the form of polysulfides (H_2_S_n_), glutathione persulfide (GSSH), glutathione trisulfide (GSSSG), cysteine persulfide (CysSSH) and so on, actually mediate some of the reported biological activities previously thought to be regulated by H_2_S^6^,^7^,^8^,^9^,^10^,^11^. So, there is increasing interest in these reactive sulfur species^5^.

So far, three H_2_S-producing enzymes have been reported: cystathionine γ-lyase (CSE), cystathionine β-synthase (CBS) and 3-mercaptopyruvate sulfurtransferase (3MST)^12^, and recently these enzymes have also been reported to produce persulfides and polysulfides^7^,^8^,^9^. CSE and CBS produce H_2_S or cysteine persulfides/polysulfides by using l-cysteine or l-cystine as a substrate, respectively, and are involved in relaxation of vascular smooth muscles and cytoprotection^1^,^7^,^8^. 3MST produces H_2_S from 3-mercaptopyruvate (3MP), which is generated from l-cysteine and α-ketoglutarate (α-KG) by cysteine aminotransferase (CAT) in the presence of the cofactors thioredoxin and dihydrolipoic acid^13^. It also produces polysulfides in the brain^9^.

To study the physiological roles and redox biology of these reactive sulfur species, we require inhibitors able to regulate the activities of the above three enzymes. Several CSE inhibitors, such as propargylglycine (PAG) and β-cyano-l-alanine (BCA), have been reported^14^,^15^, and PAG has been widely used in studies of the roles of H_2_S in mammalian physiology. Aminooxyacetic acid (AOAA) is frequently used as a CBS inhibitor. On the other hand, in the case of 3MST, only non-selective and weak substrate-like inhibitors have so far been reported^16^,^17^,^18^,^19^, and none of them is suitable for biological studies. Therefore, development of a 3MST-selective inhibitor would be extremely useful for biological studies of H_2_S and persulfides/polysulfides, and for detailed examination of the functions of 3MST.

Here, we established a novel high-throughput screening (HTS) system by utilizing our previously developed H_2_S-selective fluorescent probe, HSip-1^20^, and discovered several 3MST-selective inhibitors sharing a similar chemical structure by screening of a large chemical library (174,118 compounds) of the Drug Discovery Initiative, The University of Tokyo, Japan. Based on crystal structure determinations of 3MST complexes with two of the inhibitors, as well as theoretical calculations, we suggest that a unique long-range electrostatic interaction between the inhibitor and the persulfurated cysteine residue located in the enzyme active site plays a key role in inhibitor binding. We also discuss the implications of our findings for the reaction mechanism of the enzyme.

## Results

### Construction of HTS system for 3MST inhibitors

**Figure 1:**
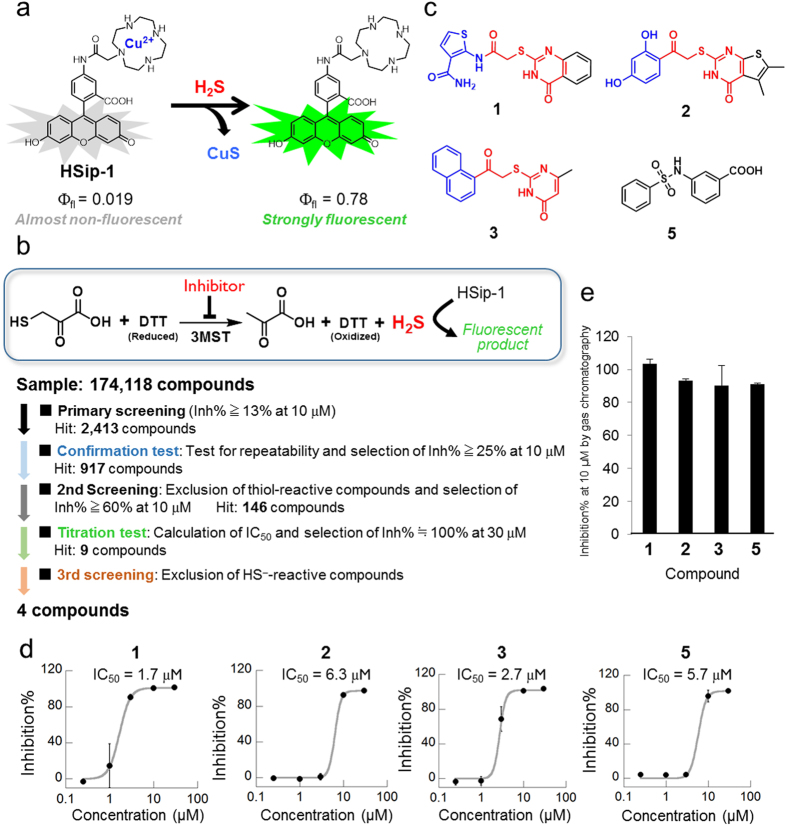
HTS scheme and hit compounds. (**a**) The fluorescent probe for H_2_S, HSip-1, and its fluorescence off/on mechanism in response to H_2_S. (**b**) Detection system of 3MST enzymatic activity with HSip-1 and the scheme for HTS of a chemical library of 174,118 compounds. (**c**) The chemical structures of 4 potential inhibitors (hit compounds) for 3MST. (**d**) Dose-response curves of inhibitory activity of compounds **1**, **2**, **3** and **5** towards 3MST in the titration test. (**e**) Inhibitory activity of hit compounds at 10 μM measured by gas chromatography.

We have previously reported a fluorescent probe for H_2_S, HSip-1, based on azamacrocyclic Cu^2+^ complex chemistry (Fig. 1a). HSip-1 can detect H_2_S in aqueous solution with high selectivity over biothiols, inorganic sulfur compounds, reactive oxygen species (ROS) and reactive nitrogen species (RNS), and has excellent photophysical properties for *in vitro* and *in cellulo* assays (Ф_fl_ = 0.019 and 0.78 in the absence and in the presence of H_2_S, respectively). Since fluorescence detection is rapid and convenient^21^, we used HSip-1 to develop an HTS system for discovery of 3MST-selective inhibitors. For this purpose, we first prepared a large amount of recombinant GST-fused 3MST. We used mouse 3MST (m3MST) because we wished to discover inhibitors that would be suitable for studies of the physiological function of 3MST in mice as model animals. We adopted an *E. coli* expression system in order to obtain a sufficient amount of GST-fused m3MST (GST-3MST) for the HTS (about 30 mg of GST-3MST from 900 mL of LB medium; Supplementary Fig. S1).

In this assay system, GST-3MST produces H_2_S by enzymatic reaction with 3MP and dithiothreitol (DTT) as substrates, and we further added HSip-1 to this solution as a fluorescent probe to monitor H_2_S production; thus, when the enzyme activity is inhibited by a test compound, and consequently the H_2_S production decreases, the fluorescence increase of HSip-1 is suppressed. First, we confirmed that HSip-1 could detect H_2_S produced by 3MST. The fluorescence intensity of HSip-1 in GST-3MST-containing reaction solution dramatically increased after addition of its substrates 3MP and DTT (Supplementary Fig. S2). On the other hand, when GST was used instead of GST-3MST as a negative control, the fluorescence increase of HSip-1 was suppressed. Thus, HSip-1 could detect H_2_S produced by 3MST in terms of a fluorescence increase. Moreover, we optimized the assay conditions in the 384-well HTS format (Supplementary Fig. S3), including appropriate concentrations of 3MST, 3MP and DTT. We also examined the effect of DMSO on this optimized enzymatic reaction of 3MST, because the library compounds are initially dissolved in DMSO as stock solutions. DMSO showed almost no effect on the enzyme activity of 3MST up to at least 5% DMSO (Supplementary Fig. S4).

### HTS of a chemical library for 3MST inhibitors

We performed 3MST inhibitor screening of a chemical library containing 174,118 compounds (Fig. 1b). All compounds were tested at 10 μM and compounds showing more than 13% inhibition were selected (primary screening; 2,417 hit compounds) (Supplementary Fig. S3 and S5). We further examined the reproducibility (confirmation test) of the hit compounds identified in the primary screening to eliminate false-positives due, for example, to dispensing errors, leaving 917 hit compounds (Supplementary Fig. S6). The second, non-enzymatic assay was then performed to eliminate false-positive hit compounds, such as naphthoquinone, showing reactivity with substrates 3MP and DTT, leaving 146 hit compounds (Supplementary Fig. S7). In the titration test, we examined the dose-dependency (0.25, 1, 3, 10, 30 μM) of 3MST-inhibitory activity of each compound (146 hit compounds in the second screening) to determine the half-maximal (50%) inhibitory concentration (IC_50_). Nine compounds (Fig. 1c, Supplementary Fig. S8) showed dose-dependent inhibition of 3MST, with IC_50_ values of 0.23–14.9 μM (Supplementary Table S1). In the third screening, we excluded false-positive compounds that directly react with H_2_S (Supplementary Table S2). For this assay, we synthesized a reported small-molecular H_2_S donor^22^ that generates H_2_S by reacting with cysteine. Based on the results of the titration test and the third screening, we concluded that compound **9** showed weak reactivity with thiol compounds such as 3MP and DTT (Supplementary Table S1), and compounds **6**–**8** appeared to react with H_2_S (Supplementary Table S2). Based on the structure, compound **9** is considered to be one of the pan-assay interference compounds (PAINS), which show activity across a range of assay platforms and against a range of proteins^23^. Compound **4** was excluded because it has a thiol group and is likely to be readily oxidized to disulfide. On the other hand, compounds **1**–**3**, **5** showed >80% inhibition of 3MST activity at 10 μM, and their IC_50_ values were 2–7 μM (Fig. 1c,d). Interestingly, **1**–**3** all have a similar structural scaffold, i.e., an aromatic ring-carbonyl-*S*-pyrimidone structure (Fig. 1c). We then confirmed the inhibitory activity of the 4 hit compounds by direct monitoring of H_2_S production by gas chromatography, because we had detected H_2_S production only with the fluorescent probe up to this point. All 4 compounds showed >80% inhibition activity at 10 μM, in agreement with the fluorescence results (Fig. 1e, Supplementary Fig. S9).

### Assessment of selectivity of hit compounds for 3MST

**Figure 2:**
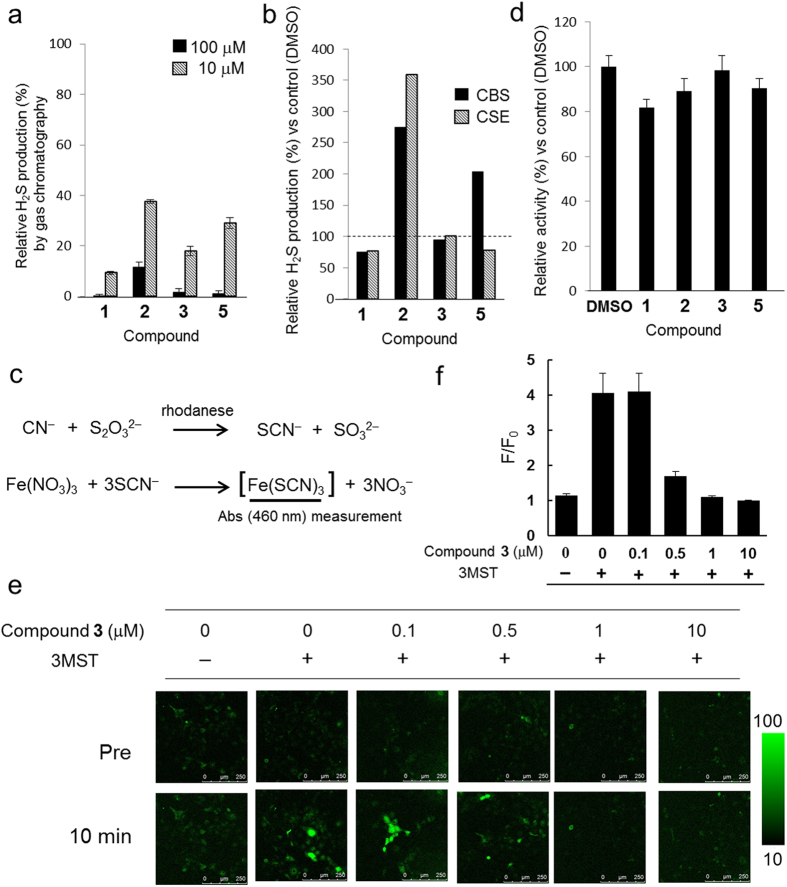
Selectivity assessment of hit compounds for 3MST over other H_2_S/polysulfides-producing enzymes and rhodanese. (**a**) 3MST-Inhibitory activity of hit compounds at 10 and 100 μM in cell lysate of 3MST-expressing HEK293 cells. 100 μM 3MP and 100 μM DTT were added to the solution containing 0.1% DMSO as a cosolvent, and the mixture was incubated at 37 °C for 15 min. H_2_S production was measured by gas chromatography. All data are presented as the mean ± S.D. (100 μM: *n* = 3 or 4; 10 μM: *n* = 3). (**b**) Selectivity assessment of hit compounds at 100 μM towards CBS and CSE. Only DMSO was added to the control sample instead of DMSO solution containing hit compounds. H_2_S production was monitored by gas chromatography. All data are presented as the mean (*n* = 3). (**c**) Detection method for rhodanese activity (Sörbo method). (**d**) Selectivity assessment of hit compounds towards rhodanese. The results are mean ± S.D. (*n* = 4, three times). These values were determined based upon the absorbance of [Fe(SCN)_3_] at 460 nm (Sӧrbo method). Bovine rhodanese 1.3–1.7 units/mL, substrate: 52 mM Na_2_S_2_O_3_, 50 mM KCN, 0.004% (w/v) BSA, 100 μM compound and DMSO 1% (v/v) in 88 mM potassium phosphate buffer (pH 8.6) at room temperature for 5 min incubation. (**e**) Fluorescence confocal microscopic images of live COS7 cells transfected with 3MST or an empty vector^9^ as a control. Cells were incubated with 50 μM SSP4, a fluorescent probe for sulfane sulfur, and 10 μM, 1 μM, 0.5 μM, 0.1 or 0 μM compound **3** in DMEM containing 0.6% DMSO for 30 min, then washed with HBSS and placed in fresh DMEM with 0‒10 μM compound **3** (Pre). 500 μM 3MP was added to the cells and the cells were incubated for 10 min (10 min). (**f**) Graphic representation of (**e**). F/F_0_ was calculated by dividing the fluorescence intensity of cells after addition of 500 μM 3MP (10 min) by that of cells before addition of 500 μM 3MP (Pre), then the mean F/F_0_ was determined from 5 cells. All data represent the mean ± standard error of the mean (SEM) of three experiments.

To examine the selectivity of these compounds for 3MST, we measured their inhibitory activity in cell lysate of 3MST-overexpressing HEK293 cells by gas chromatography (Fig. 2a). All 4 compounds showed >85% inhibition at 100 μM, and compounds **1** and **3** showed high inhibitory activity (80–90%) even at 10 μM. We further examined the selectivity of compounds **1**–**3** and **5** against the other two H_2_S-producing enzymes, CSE and CBS by gas chromatography. Compound **1** showed about 25% inhibition of recombinant CBS and CSE enzymatic activities, while compound **3** was almost inactive towards CBS and CSE (Fig. 2b). Interestingly, compound **2** enhanced H_2_S production by CBS and CSE, but this is possibly due to the direct reaction of **2** with the high concentration of cysteine (10 mM). Compound **5** showed enhancement of H_2_S production by CBS only. The mechanisms of enhanced H_2_S production by compounds **2** and **5** are under investigation. Finally, we examined the inhibitory activity of the 4 hit compounds towards rhodanese (thiosulfate sulfurtransferase, Type II), which belongs to the same rhodanese/Cdc25 phosphatase superfamily as 3MST^24^ and shares high amino acid sequence identity with m3MST (58%; 172/297 amino acids). This enzyme is involved in cyanide metabolism by transferring a sulfur atom to cyanide ion. We monitored the conversion of cyanide ions to thiocyanide ions by rhodanese using the Sӧrbo method^25^ (Fig. 2c, Supplementary Fig. S10), and found that compounds **1**, **2**, **3** and **5** showed 18.2 ± 3.6%, 10.9 ± 5.5%, 1.7 ± 6.7% and 9.7 ± 4.5% inhibition of the conversion at 100 μM, respectively (Fig. 2d). Thus, compound **3** showed the highest selectivity for 3MST. Next, we applied compound **3** to 3MST-overexpressing COS7 cells to examine its suitability for live cell experiments. 3MST activity in living cells was almost completely suppressed by 1 μM **3**, demonstrating that **3** is cell-membrane permeable and should be suitable for use in biological studies (Fig. 2e,f).

### X-Ray crystal structure determination of 3MST-inhibitor complexes

**Figure 3:**
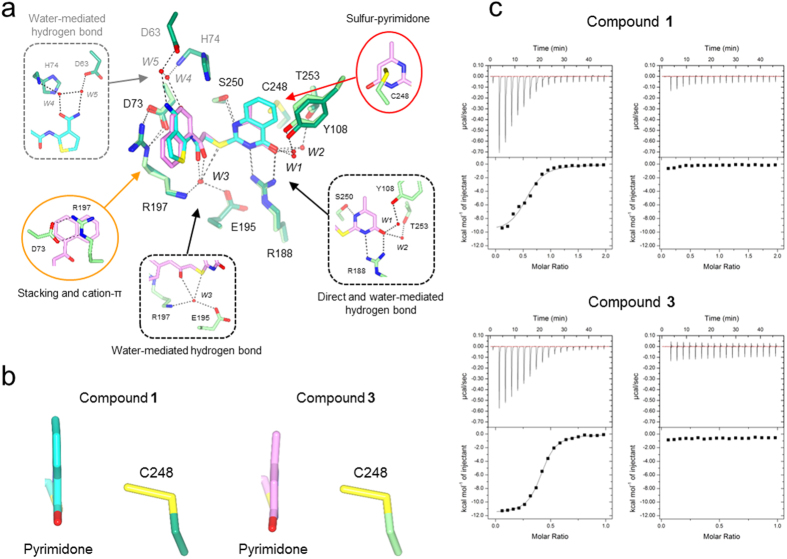
Crystal structure of 3MST-inhibitor complex and ITC analysis. (**a**) Detailed view of the active site of the two protein-inhibitor complexes. Residues of 3MST-compound **1** complex (emerald green), residues of 3MST-compound **3** complex (light green), compound **1** (cyan), compound **3** (pink), oxygen atoms of residues and compounds (red), nitrogen atoms (blue), sulfur atoms (yellow), hydrogen bonding (black dotted lines), waters of 3MST-compound **1** complex (red spheres) and waters of 3MST-compound **3** complex (red spheres) are shown. (**b**) Detailed view of the 4-pyrimidone-like aromatic ring and the side chain of persulfurated C248. The side chain of 3MST-compound **1** complex (emerald green), the side chain of 3MST-compound **3** complex (light green), compound **1** (cyan), compound **3** (pink), oxygen atoms (red), nitrogen atoms (blue) and sulfur atoms (yellow) are shown. (**c**) ITC analysis of 3MST injected with compound **1** (upper) and compound **3** (bottom). The titration plots (top graph) and fitting curves (bottom graph) of persulfurated 3MST (left) and the non-persulfurated 3MST (right) are shown.

We determined the crystal structures of the m3MST complexes with **1** and **3** at high resolution (Supplementary Table S3). The most remarkable feature is that C248 is persulfurated in both crystal structures (Fig. 3a,b). Persulfurated C248 appears to interact with the 4-pyrimidone-like aromatic ring of both compounds (Fig. 3b). To our knowledge, this interaction is novel in terms of both the long distance (>3.45 Å) and the orientation of the persulfide bond (almost perpendicular to the aromatic ring of the inhibitors). Direct hydrogen bonding by R188 and S250 and water-mediated hydrogen bonding by E195 and R197 are commonly observed with compounds **1** and **3**. In addition, compound **1** forms water-mediated hydrogen bonds with D63 and H74. Both compounds were in van der Waals contact with W36, L38, P39, D73, H74, Y108, R188, P196, R197, G249, S250, V252 and V277. The thiophene ring (compound **1**) and naphthalene ring (compound **3**) exhibit cation-π interaction with R197 and parallel stacking interaction with the salt bridge between D73 and R197^26^.

To examine the importance of the persulfurated Cys residue, we further performed ITC measurement to determine the dissociation constant *K*_d_ between 3MST and compounds **1** and **3**. Heat release was detected during mixing of the inhibitor and persulfurated 3MST, and the dissociation constants were calculated to be 3.0 μM (compound **1**) and 0.5 μM (compound **3**) (Fig. 3c, left). The persulfurated or non-persulfurated 3MST is the treated or non-treated 3MST with the substrate, 3MP, respectively (The details are described in Methods). The Cys248 existed as un-persulfurated form in crystal structure of 3MP-untreated 3MST (data not shown). In contrast, no heat release or UV-vis absorption change (data not shown) was observed when non-persulfurated 3MST was used (Fig. 3c, right). Thus, it is considered that the persulfurated C248 residue of 3MST is necessary for stable complexation with the inhibitors.

### Computational insight into the interaction between the pyrimidone ring and persulfurated cysteine residue

**Figure 4:**
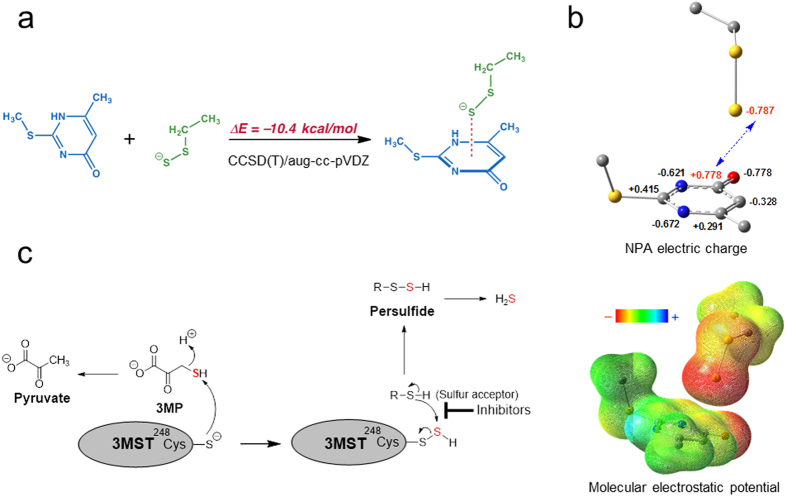
Molecular orbital calculation of interaction energy between persulfided cysteine residue and inhibitors. (**a**) Interaction energy calculation of model compounds by CCSD-T/AUG-CC-PVDZ. Calculated interaction energy was −10.4 kcal/mol and this interaction is highly stabilized. (**b**) Charge distribution of model persulfided molecule and pyrimidone structure. Strong electrostatic interaction was observed between these model molecules. (**c**) The putative enzymatic reaction mechanism of 3MST (the ping-pong mechanism) is shown. Persulfide or H_2_S is generated from 3MP via 3MST. The inhibitor found in this study blocks the second step of the enzymatic reaction, i.e., the transfer of a sulfur atom from the persulfurated cysteine residue at active site to an sulfur acceptor.

We performed theoretical calculations to examine the interaction of the pyrimidone structure of the inhibitors and the persulfurated cysteine residue of 3MST, using high-level coupled-cluster calculation (CCSD(T)) combined with a large aug-cc-pVDZ basis set. The model pyrimidone and S–S^−^ molecules were positioned based on the X-ray crystal structure (Fig. 4a). Although crystallographic structures sometimes exaggerate electrostatic interactions, these structures were considered to be a reasonable starting point for consideration of the unprecedented interaction. Gas-phase calculations indicated that the interaction of S–S^−^ with pyrimidone is strongly exothermic (10.4 kcal/mol; CCSD(T)/aug-cc-pVDZ), which is not inconsistent with the experimental ITC measurements (Fig. 3c; ΔH = −9.37 and −11.76 kcal/mol for compounds **1** and **3**, respectively). The stabilization energy is very large compared to hydrogen bonds involving amino acids (ca. 1–5 kcal/mol)^27^. To understand this strong interaction, we then performed NBO (Natural Bond Orbital) analysis of the donor-acceptor interaction between the S–S^−^ and the pyrimidone moiety. The S–S^−^ residue is regarded as a potent nucleophile, because the S^−^ anion is strongly activated by the α-effect of the lone pair of the adjacent sulfur atom. Indeed, the persulfurated cysteine residue is highly reactive and nucleophilic addition occurs with various electrophilic moieties (molecules). However, NBO analysis of the complex indicated no large stabilization interactions (<1 kcal/mol, Supplementary Table S4 and Table S5), because the distance between the S^−^ atom and the pyrimidone ring is too far for orbital interactions. We concluded that the interaction is predominantly due to electrostatic attraction between the persulfur anion and the positively charged carbonyl carbon of the pyrimidone structure (Fig. 4b).

## Discussion

To date, no specific inhibitor of 3MST has been reported. However, application of our recently developed H_2_S-selective fluorescent probe for HTS of a large chemical library enabled us to discover several inhibitors. Notably, inhibitors **1** and **3** do not bind to non-persulfurated 3MST, but bind to an intermediate persulfurated 3MST as determined by ITC and X-ray crystallographic analysis. Thus, we concluded that both compounds inhibit the sulfur transfer reaction from the persulfurated intermediate by blocking the binding of the sulfur acceptor to 3MST (Fig. 4c). 3MST has recently been reported to produce not only H_2_S, but also H_2_S_n_^9^, and our inhibitors also block the production of H_2_S_n_ by 3MST (Fig. 2e). It has been suggested that 3MST catalyzes the cleavage of a carbon-sulfur bond and the transfer of a sulfur atom from 3MST to any of a variety of thiophiles, including thiols, cyanides, sulfite, and sulfinates via a sequential mechanism, i.e., the mechanism of the catalysis has been mostly presumed to be a double displacement via the ternary complex of 3MST, 3MP and thiophiles unlike rhodanase^28^,^29^. Our crystal structure determination shows that the 3MP-binding site of 3MST is too small to allow simultaneous binding of 3MP and a thiol, and the 3MST–inhibitor complexes contained the inhibitor without pyruvate (metabolite of 3MP), presumably because the pyruvate was released from 3MST before the inhibitor binds. Thus, our work provides the experimental support that enzymatic catalysis of 3MST with 3MP proceeds via a ping-pong mechanism, like NAD(P)H-related enzymatic reactions^30^; i.e., the mechanism of the catalysis is a double displacement involving initial formation of the sulfur-substituted (persulfurated cysteine residue-containing) enzyme, as well as rhodanase (Fig. 4c).

It has been reported that the Michaelis constant *K*_m_ of 3MP for 3MST is 4.08 mM^15^ and this affinity is relatively weak. Some 3MP(substrate)-like inhibitors of 3MST, such as pyruvate and 3-mercaptopropionic acid, also show inhibition constant *K*_i_ values of several mM^15^,^17^. Nevertheless, in this study, we discovered several relatively potent inhibitors of 3MST with *K*_d_ of μM order, and we concluded that this was due to a strong electrostatic interaction between inhibitor and the persulfurated cysteine residue. X-Ray studies have shown that some other enzymes contain a per/polysulfurated cysteine residue in the active site^31^,^32^,^33^,^34^,^35^,^36^,^37^, so the findings in this study may also be useful for the molecular design of inhibitors targeting these enzymes.

Our computations indicated that the strong interaction between the persulfurated cysteine residue and the pyrimidone-like structure of the inhibitors is electrostatic in nature. This idea is consistent with the relatively long distance between the two moieties, and is also supported by the calculated interaction energy ∆*E* between the lone pairs of the sulfur atom and the LUMO of the pyrimidone moiety. Some groups have reported aromatic‒thiol π‒type hydrogen bonding^38^,^39^, but the calculated ∆*E* between the lone pairs of the sulfur atom and the LUMO of the pyrimidone moiety is too small, ruling this out in the present case. We suspect that this interaction is very sensitive to the orientation of the two lone electron pairs on the sulfur atom relative to the π electron cloud of the aromatic ring, so that interaction configurations that provide significant bonding energy exist only within a narrow configurational space. Thus, in our best knowledge, this strong interaction has never been observed before.

m3MST and human 3MST (h3MST) share high sequence identity (84.5%; 251/297 amino acids). m3MST has two rhodanese-like domains (residues 1–138 and 165–297, according to h3MST numbering^40^) (Supplementary Fig. S11), and the active site is positioned between them. The overall structure of m3MST in the complexes is quite similar to that of h3MST (r.m.s.d. 0.62–0.84 Å) (PDB ID: 3OLH, 4JGT)^40^, but there are several differences between m3MST and h3MST in the active site (Supplementary Fig. S12). One is the position of R197. The side chain of R197 of h3MST is positioned inside the active site and forms a hydrogen bond with pyruvate, whereas that of m3MST-inhibitor complexes is directed outside of the active site and forms a hydrogen bond with D73. The arginine residues in the substrate binding site have been reported to be critical residues in determining substrate specificity for 3MST^41^, and this difference may be due to the binding molecule, i.e., compound **3** and pyruvate. Other side chains are also located at somewhat different positions. Therefore, it is likely that the chemical structures of compounds **1** and **3** will need to be tuned to create selective inhibitors of h3MST.

In summary, we constructed an HTS system with easy-to-use fluorescence detection, utilizing our selective fluorescent probe for H_2_S, HSip-1, and discovered several selective inhibitors of 3MST by screening a library of 174,118 compounds. We analyzed the binding mode between the inhibitors and 3MST. Interestingly, the main interaction was an unprecedented electrostatic interaction between the persulfurated cysteine residue of 3MST and the pyrimidone structure of the inhibitors. Persulfurated cysteine can exist in biological environments^7^,^8^,^9^, and crystal structure determination of several enzymes having the persulfurated cysteine residue indicates that in most cases, the residue is positioned in the active site. Therefore, it may be possible to utilize a similar electrostatic interaction in the molecular design of inhibitors for those enzymes as well. The calculated p*K*_a_ of cysteine persulfide is 4.34 and the persulfurated cysteine residue is thought to be in a deprotonated anionic form^42^, which would be consistent with the strong electrostatic interaction found in this study. Further, our analysis of the binding mode between the inhibitors and 3MST has provided the experimental support for the ping-pong enzymatic mechanism of 3MST with 3MP as a substrate. On the other hand, 3MST activity is elevated in human neoplastic cell lines^43^ and erythrocytes from patients with polycythemia vera^44^, so selective 3MST inhibitors might have therapeutic value for these diseases. 3MST-knockout mice were recently reported as a model for human mercaptolactate-cysteine disulfiduria^45^ and these mice should be a useful tool for investigating the physiological roles of 3MST. The selective 3MST inhibitors discovered in the present study should also be invaluable for *in vitro* and *in vivo* studies along this line.

## Methods

### HEK293 cell culture

HEK293 cells were cultured in Dulbecco’s modified Eagle’s medium (DMEM (Wako, 044–29765)) supplemented with 10% (v/v) fetal bovine serum, penicillin (100 units/mL), and streptomycin (100 μg/mL) in a humidified incubator under 5% CO_2_ in air.

### Preparation of recombinant 3MST

The constructed m3MST (UniProt ID: Q505N7)/pGEX and pGEX^46^ were each transformed into *E. coli* BL21 cells. The transformed *E. coli* cells were grown overnight 1 mL 2 × YT medium in the presence of 100 μg/mL ampicillin (small scale culture) at 37 °C, then diluted with 150 mL of LB medium in the presence of 100 μg/mL ampicillin, and cultured to OD_600_~0.9–1.3 (3MST 1.1 or 1.2, GST 1.0 or 1.3) at 37 °C (large-scale culture). IPTG (final 0.1 mM) was added to the culture medium to induce expression of GST-3MST or GST, and culture was continued for 21 hr at 20  °C. Cells were harvested by centrifugation (3000 rpm, 4 °C) for 15 min and resuspended in 12.5 mL of D-PBS buffer (2.7 mM KCl, 137 mM NaCl, 1.5 mM KH_2_PO_4_ and 8.1 mM Na_2_HPO_4_) supplemented with lysozyme, 1% Triton X, 10 U DNase (Benzonase, Novagen), 1 mM DTT, and 1% protease inhibitor cocktail (Sigma) on ice for 60 min. The lysate was sonicated for 10 sec three times on ice and centrifuged (3000 rpm, 4 °C) for 15 min to obtain the soluble fraction. The supernatant was purified with a GST purification module (GST GraviTrap, GE Healthcare), and desalted with a PD-10 column (GE Healthcare) according to the manufacturer’s instructions. The elution buffer was D-PBS. The collected fractions were analyzed by SDS-PAGE (4–20% gradient acrylamide).

### Bradford assay for protein quantification

Dye reagent was prepared by diluting 1 part dye reagent (Bio-Rad) concentrate with 4 parts Milli-Q water. The solution was filtered through a Millipore Millex-LH 0.45 μm (Millipore) to remove particulates. 10 μL each of standard (BSA) and sample solution were added to 200 μL of diluted dye reagent in a 96-well plate, and vortex-mixed. The plate was incubated at room temperature for 10 min and the absorbance of each well was measured at 595 nm with a microplate reader (*n* = 4).

### 3MST inhibitor screening (Primary screening, confirmation test)

High-throughput screening (HTS) was performed with a chemical library (174,118 compounds at 1st screening and 2,417 compounds at confirmation test) from the Drug Discovery Initiative, The University of Tokyo. Screening methods were as follows: first, low-molecular-weight compounds (1 mM DMSO solution, 200 nL) from library plates were diluted with 10 μL of HSip-1 and 3MST solution to 20 μM (2.0% DMSO) with a Multi-Drop Combi (Thermo) (320 compounds (*n* = 1) or 80 compounds (*n* = 4)/384-well plate). Next, a solution of 3MP and DTT (10 μL) was dispensed into the plates (total volume was 20.2 μL), which were incubated at room temperature for 3 hr. To assay a lot of 384-well plates simultaneously, we set the reaction time to 3 hours. The fluorescence of HSip-1 was measured (λ_ex_ = 485 nm, λ_em_ = 520 nm) with a microplate reader (PHERAstar PLUS (BMG LABTECH)). Background control wells (i.e., GST wells instead of GST-3MST, *n* = 16) were also prepared for all plates (see Supplementary Fig. S3).

### Reactivity test toward 3MP and DTT (2nd screening)

Reactivity of the hit compounds toward 3MP and DTT was examined. The final concentration of the compounds was 10 or 50 μM. The assay protocol was as follows: compounds were diluted with HSip-1 solution to 20 or 100 μM solution (10% DMSO) with a Multi-Drop Combi. Next, 3MP and DTT solution (10 μL, final concentrations: 50 μM 3MP and 40 μM DTT) was dispensed into the plates (total volume was 20 μL, 5% DMSO), and the plates were incubated at room temperature for 3 hr. The fluorescence of HSip-1 was measured (λ_ex_ = 485 nm, λ_em_ = 520 nm) with a microplate reader (PHERAstar PLUS). Background control wells (i.e., no addition wells instead of addition of 3MP and DTT, *n* = 16) were also prepared for all plates.

### 3MST inhibitor screening (Titration test)

The dose-dependency of 3MST inhibition by 146 hit compounds from the second screening was examined to determine the IC_50_ values. Screening methods were as follows: first, low-molecular-weight compounds (2 mM DMSO stock solution) from library plates (the final concentrations of the compounds were 0.25, 1, 3, 10, 30 μM) were diluted with HSip-1 and 3MST solution to 20 μM solution (10% DMSO) with a Multi-Drop Combi. Next, a solution of 3MP and DTT (10 μL) was dispensed into the plates (total volume was 20 μL, 5% DMSO), and the plates were incubated at room temperature for 3 hr. The fluorescence of HSip-1 was measured (λ_ex_ = 485 nm, λ_em_ = 520 nm) with a microplate reader (PHERAstar PLUS). Background control wells (i.e., GST wells instead of GST-3MST, *n* = 16) were also prepared for all plates.

### Reactivity test toward H_2_S (3rd screening)

Reactivity of the hit compounds toward H_2_S was next examined. The final concentration of the compounds was 10 or 30 μM. The assay protocol was as follows. Compounds were diluted with HSip-1 and cysteine-activated H_2_S donor^22^ solution to 20 or 60 μM solution (15.4% DMSO) with a Multi-Drop and a pipette. Next, 2 mM l-cysteine solution (10 μL, final concentration: 1 mM l-cysteine) was dispensed into the plates (total volume was 20 μL, DMSO 7.7%), and the plates were incubated at 37 °C for 1 hr. The fluorescence of HSip-1 was measured (λ_ex_ = 490 nm, λ_em_ = 510 nm) with a microplate reader. Background control wells (i.e., no addition wells instead of addition of the cysteine-activated H_2_S donor, *n* = 16) were also prepared for all plates.

### Determination of inhibitory activity of hit compounds toward purified m3MST by means of gas chromatography

1 μL of 1 mM compound (final 10 μM) was diluted with 89 μL of 3MST solution (final 3.8 μg/mL) or GST solution (2.2 μg/mL) in 30 mM HEPES buffer (pH 7.4) in a 30 mL collection vial (Iwaki). 10 μL of 3MP and DTT solution (final concentration: 50 μM 3MP and 40 μM DTT) was added to 90 μL of the mixture, then the vial was sealed with parafilm and incubated at room temperature for 3 hr. After addition of 200 μL of 1 M sodium citrate buffer (pH 6.0), the mixture was incubated at 37 °C for at least 10 minutes with shaking on rotary shaker to quench the enzymatic activity and facilitate release of H_2_S gas from the aqueous phase. A 2 mL sample of head-space gas (total, approximately 29.5 mL) was applied to a gas chromatograph (ODSA-P2, Alpha M.O.S. Japan). The concentrations of H_2_S were determined from a standard curve obtained with 0 to 4 nmol of Na_2_S, which is a source of H_2_S. The protocol is illustrated in Supplementary Fig. 9.

### Transient transfection of HEK 293 cells

Transient transfection of HEK 293 cells was performed with *Trans*IT-LT1 (Takara). For transfection, 14 μg of expression plasmid (3MST/pCI)^9^ was mixed with 45 μL of *Trans*IT-LT1 in 1.5 mL of Opti-MEM (Invitrogen) and then added to HEK 293 cells at 60–70% confluency in a 9 cm dish. Cells were harvested at 48 h post-transfection and washed with ice-cold PBS. After precipitation by centrifugation, cell pellets were resuspended in the ice-cold buffer and sonicated.

### Determination of inhibitory activity of the lysate of m3MST-overexpressing cells by means of gas chromatography

HEK 293 cells in ice-cold isolation buffer containing 30 mM HEPES buffer (pH 7.4), 100 μM DTT and 1% protease inhibitor cocktail (Sigma, Japan) were sonicated for 10 sec twice in an Astrason 4000 sonicator (MISONIX, USA) to obtain the lysate. Protein concentration was determined by use of the Bio-Rad Bradford Protein Assay (Bio-Rad Laboratories) according to the manufacturer’s instructions. The enzyme reaction was performed as reported, with some modifications^46^,^47^. 1 μL of 10 mM compound (final 100 μM) was diluted with 89 μL of lysate in a 30 mL collection vial (Iwaki). 10 μL of a mixture of 1 mM 3MP (final 100 μM) and 100 μM DTT was added to 90 μL of the cell lysate, and the vial was sealed with parafilm and incubated at 37 °C for 15 min. After addition of 200 μL of 1 M sodium citrate buffer (pH 6.0), the mixture was incubated at 37 °C for at least 10 minutes with shaking on a rotary shaker to quench the enzymatic activity and facilitate the release of H_2_S gas from the aqueous phase. A mixture of 1 mL of head-space gas (total, approximately 29.5 mL) and 1 mL of air was applied to a gas chromatograph (ODSA-P2, Alpha M.O.S. Japan). The concentrations of H_2_S were determined from a standard curve obtained with 0 to 8 nmol of Na_2_S, which is a source of H_2_S.

### Construction of rat CSE-HA and rat CBS expression plasmids

Rat CSE or CBS cDNA^7^ was digested with restriction enzymes (XhoI/NotI for CSE, EcoRI/NotI for CBS) and ligated into the XhoI/NotI (CSE) or EcoRI/NotI (CBS) site of the pGEX-6P vector (GE Healthcare) using a Rapid DNA Ligation Kit (Roche Applied Science). The nucleotide sequences of rCSE-HA and rCBS were confirmed by use of an Applied Biosystems 3130 Genetic Analyzer.

### Expression and purification of recombinant rat CSE-HA and rat CBS-HA

GST-tagged rat CSE-HA or rat CBS-HA was expressed in *E. coli* DH5α as follows. *E. coli* DH5α transformed with the pGEX-rCSE-HA or pGEX-rCBS-HA plasmid was cultured overnight in LB medium containing 100 μg/mL ampicillin (and 0.3 mM 5-aminolevulinic acid, for CBS) at 37 °C, and the 120 mL culture was inoculated into 2400 mL of LB medium. Further growth of the culture was performed at 37 °C with vigorous shaking until OD600 reached 0.8. IPTG was added to the *E. coli* DH5α culture at a final concentration of 0.1 mM and incubation was continued at 18 °C. After 24 h, *E. coli* DH5α was collected by centrifugation, washed with cold TBS, and lysed in 120 mL of purification buffer (50 mM Tris-HCl pH7.5, 150 mM NaCl, 0.2 mM EDTA, 0.2 mM EGTA, 0.5 mM PMSF, 1 mM DTT) by adding 1 mg/mL lysozyme, 1% Triton X-100 (final concentration), and 50 units TurboNuclease (Nacalai Tesque) on ice for 1 h followed by sonication. After centrifugation at 15000 × g for 60 min, the supernatant was applied to a 2 mL of COSMOGEL GST-Accept (Nacalai Tesque) and washed with 30 mL of purification buffer. The fusion protein was cleaved on the column with 800 μL of purification buffer containing 40 units of Turbo3C Protease (Nacalai Tesque) for 15 h at 4 °C. The pass-through fraction was collected and stored at −30 °C after addition of an equal volume of a solution containing 80% glycerol and 20% ethylene glycol.

### Determination of inhibitory activity towards purified CBS by means of gas chromatography

1 μL of 10 mM compounds (final 100 μM) was diluted with 89 μL of CBS solution (final 16.4 μg/mL) in 30 mM HEPES buffer (pH 7.4) containing PLP (final 50 μM) and SAM (final 100 μM) in a 15 mL centrifuge tube. 10 μL of l-cysteine and dl-homocysteine solution (final 10 mM l-cysteine and dl-homocysteine) was added to 90 μL of the mixture, then the tube was sealed with parafilm and incubated at 37 °C for 15 min. After addition of 200 μL of 1 M sodium citrate buffer (pH 6.0), the mixture was incubated at 37 °C for at least 10 minutes with shaking on a rotary shaker to quench the enzymatic activity and facilitate the release of H_2_S gas from the aqueous phase. A 600 μL sample of head-space gas (total, approximately 14.5 mL) was applied to a gas chromatograph equipped with a flame photometric detector and a data processor C-R8A Chromatopac (Shimadzu). The concentrations of H_2_S were determined from a standard curve obtained with 0 to 5 nmol of Na_2_S, which is a source of H_2_S.

### Determination of inhibitory activity towards purified CSE by means of gas chromatography

1 μL of 10 mM compounds (final 100 μM) was diluted with 89 μL of CSE solution (final 6.0 μg/mL) in 30 mM HEPES buffer (pH 7.4) containing PLP (final 50 μM) in a 15 mL centrifuge tube. 10 μL of l-cysteine solution (final 10 mM l-cysteine) was added to 90 μL of the mixture, then the tube was sealed with parafilm and incubated at 37 °C for 15 min. After addition of 200 μL of 1 M sodium citrate buffer (pH 6.0), the mixture was incubated at 37 °C for at least 10 minutes with shaking on rotary shaker to quench the enzymatic activity and facilitate the release of H_2_S gas from the aqueous phase. A 1 mL sample of head-space gas (total, approximately 14.5 mL) was applied to a gas chromatograph equipped with a flame photometric detector and a data processor C-R8A Chromatopac (Shimadzu). The concentrations of H_2_S were determined from a standard curve obtained with 0 to 5 nmol of Na_2_S, which is a source of H_2_S.

### Live-cell fluorescence imaging experiment

COS7 cells, purchased from ATCC (American Type Culture Collection, USA), were cultured in DMEM (Dulbecco’s modified Eagle’s medium) (Gibco 11885), containing 10% fetal bovine albumin (Invitrogen) and 1% penicillin streptomycin (Invitrogen). Cells were maintained at 37 °C under an atmosphere of 5% CO_2_ in air. Transient transfection of COS7 cells was performed with Lipofectamine LTX and PLUS reagents (Invitrogen). As the standard condition, 0.7 μg plasmid^9^, 0.7 μL PLUS reagent, and 2 μL lipofectamine LTX were mixed in 100 μL Opti-MEM (Gibco) and 5 μL of the mixture was added to the cells (0.2 mL of the medium). Transfection efficiency was confirmed to be 30–45% using pHcRed1-Nuc Vector (Clontech) as a control. COS7 cells seeded on 1 μ-Slide 8-well IbiTreat (Ibidi) were washed with 200 μL of Hanks’ Balanced Salt Solutions (HBSS) 18 hr after transfection, and then incubated for 30 min at 37 °C in 200 μL DMEM (Gibco 21063) containing 50 μM SSP4 (a commercially available fluorescent probe for sulfane sulfur (DOJINDO, Japan)) and 0–10 μM inhibitor with 0.6% DMSO and 0.03% Pluronic F127 as a cosolvent. After incubation, cells were washed twice with HBSS and 200 μL DMEM (Gibco 21063) containing 0–10 μM inhibitor with 0.1% DMSO as a cosolvent was added. Incubation was continued in DMEM containing 500 μM 3-mercaptopyruvate (3MP) for 10 min at 37 °C. Fluorescence images were captured using a Leica Application Suite Advanced Fluorescence (LAS-AF) with a TCS SP5 and a 40× objective lens. The light sources were an argon laser and white light laser. The excitation wavelengths were 488 nm and 591 nm. The emission wavelengths were 500–540 nm for SSP4 (PMT; Gain: 1000) and 600–650 nm for RFP (HyD; Gain: 100).

### Gene expression and protein purification for ITC analysis and X-ray crystallography

The recombinant full-length wild-type mouse 3MST and mutant m3MST (C65S/C255S/C264S) were expressed as an N-terminal His-tagged protein (MGSSHHHHHHSSGLEVLFQGPGS-3MST) in *E. coli* JM109 (DE3). The pET44a(+) vector (Novagen) was used as an expression vector. Cells were grown at 37 °C in LB broth (Miller) or 2 × YT broth containing 0.1 g/L ampicillin to OD_600_ about 0.5, and then protein expression was induced by adding IPTG (final conc. 0.5 mM (LB), 1 mM (2 × YT)). Cells were cultured overnight at 18–20 °C with shaking and harvested at 4 °C by centrifugation at 9,000 rpm for 5 min. Harvested cells were resuspended in lysis buffer containing 0.1 M HEPES-NaOH pH 8, 0.5 M NaCl, 0% or 10% glycerol, 0.5 mM TCEP, 1 mM PMSF, and 1% EtOH, and disrupted by sonication. Cell lysis solutions were centrifuged at 20,000 rpm for 20 min. Mouse 3MST proteins were affinity-purified on cOmplete His-Tag Purification Resin (Roche) using buffer containing 0.02 M HEPES pH 8, 0.15 M NaCl, 10% glycerol, 0.5 mM TCEP, 0–0.25 M imidazole. The proteins were further purified on HisTrap HP (GE Healthcare) by increasing the imidazole concentration in buffer containing 0.02 M HEPES pH 8, 0.15 M NaCl, 10% glycerol, and 0.05 mM TCEP until proteins were eluted. Then the His-tag was removed by GST-fusion HRV 3 C protease digestion. The proteins were further purified by anion exchange chromatography on a HiTrap Q HP (GE Healthcare) by increasing the NaCl concentration in buffer containing 0.02 M HEPES pH 8, 10% glycerol, 0.5 mM TCEP until proteins were eluted, and then by gel filtration chromatography on Superdex 75 prep grade using buffer containing 0.02 M HEPES pH 7.5, 0.3 M NaCl, 0.5 mM TCEP. Wild-type 3MST was used in ITC analysis and mutant 3MST was used for crystallization.

### ITC analysis

Persulfurated 3MST was prepared as follows: 3MST-3MP solution (5 μM 3MST, 25 μM 3MP, 20 μM TCEP, 0.02 M glycine-NaOH pH9, 0.1 M NaCl) was incubated for 2 hr at room temperature. To prevent buffer mismatch and to remove unreacted 3MP and reaction product (pyruvate), the reaction solution was exchanged to ITC measurement solution (0.02 M glycine-NaOH pH 9, 0.1 M NaCl, 5% DMSO) using an Amicon Ultra (10 K) (Millipore) or Vivaspin (10 K) (Sartorius). Non-persulfurated 3MST solution was also prepared by buffer exchange. Both inhibitors were dissolved in the ITC measurement solution. Inhibitor solution (0.5 mM compound **1** or 0.25 mM compound **3**) were titrated into a protein solution (0.05 mM 3MST) at 25 °C using a MicroCal iTC200 (GE Healthcare). The first injection of 0.4 μL was followed by 18 injections of 2 μL with 150 s between each injection. Each experiment was repeated three times (*n* = 3). For data analysis, the first injection peak was removed. Data analyses and curve fitting to a single-site binding model were performed by Origin7 SR4 software (OriginLab).

### Crystallization and structure determination

The concentrations of protein-inhibitor complexes during crystallization could not be precisely determined because we did not evaluate the inhibitor binding rate, so the following final concentrations are estimated values. 3MST-compound **1** complex was prepared as follows. Protein-compound **1** solution (0.08 mg/mL or 0.17 mg/mL 3MST, 10 μM compound **1**, 50 μM 3MP, 0 μM or 40 μM TCEP, 0.03 M glycine-NaOH pH 9, 0.1 M NaCl, 1% DMSO) was incubated overnight at 25 °C. The m3MST-compound **1** complex solution was exchanged to a crystallization solution (0.02 M glycine-NaOH pH 9, 0.3 M NaCl) and concentrated to about 10–30 mg/mL using an Amicon Ultra (10 K). 3MST-compound **3** complex was prepared as follows. Protein-compound **3** solution (0.08 mg/mL 3MST, 10 μM compound **3**, 50 μM 3MP, 40 μM TCEP, 0.03 M HEPES-NaOH pH 8.2, 0.1 M NaCl, 1% DMSO) was incubated overnight at 25 °C. The 3MST-compound **3** complex solution was also exchanged to a crystallization solution (0.02 M HEPES-NaOH pH 8.2, 0.3 M NaCl) and concentrated to about 10–30 mg/mL using an Amicon Ultra (10 K). Crystals were grown at 4 °C by the sitting-drop vapor diffusion method by mixing protein-inhibitor complex solution and reservoir solution (36% (w/v) PEG 3,350, 0.1 M HEPES-NaOH pH 8.1) in a volume ratio of 4:1, and equilibrating the samples against 50 μL of reservoir solution. Crystals of 3MST-compound **3** complex often appeared in or around precipitates that were generated during preparation of the complex. The crystals used for data collection were cryo-cooled in liquid nitrogen using reservoir solution as a cryoprotectant. X-Ray diffraction data were collected at 100 K at the PF/KEK BL5A beamline and at the SPring-8 BL44XU beamline. Diffraction data were integrated and scaled using the program HKL2000 (HKL Research, Inc.)^48^. The initial phases were determined by molecular replacement with the program Molrep^49^ using h3MST (PDB ID: 3OLH) or the determined m3MST structure as a search model. Model buildings were performed with the program WinCOOT^50^,^51^. Structural refinements were performed with the programs Refmac5^52^,^53^ and PHENIX^54^. After several cycles of manual model building and structural refinement, the electron density corresponding to the inhibitor was clearly observed. Chemical structures of both inhibitors were drawn using ChemSketch^55^ (Advanced Chemistry Development, Inc. (ACD/Labs)), and then regularization and chemical restraint parameters for structural refinement were generated using JLigand^56^. 3MST-compound **1** complex was refined using intensity-based twin refinement with anisotropic temperature factors for non-hydrogen atoms. The geometry of the structure was verified using the PROCHECK program^57^. Coordinates and the structure factors were deposited in the Protein Data Bank (PDB code: 5WQJ and 5WQK).

### Calculation methods

All calculations were performed with the Gaussian 09 program system (Revision D.01)^58^. Single-point energy was calculated in the gas phase based on the single crystal structure at the CCSD(T) level^59^ with the aug-cc-pVDZ basis set^60^,^61^. NBO (Natural Bond Orbital) calculation was performed at the same level with the NBO 3.1 package in the Gaussian 09 program.

## Additional Information

**How to cite this article**: Hanaoka, K. *et al*. Discovery and Mechanistic Characterization of Selective Inhibitors of H_2_S-producing Enzyme: 3-Mercaptopyruvate Sulfurtransferase (3MST) Targeting Active-site Cysteine Persulfide. *Sci. Rep.* **7**, 40227; doi: 10.1038/srep40227 (2017).

**Publisher's note:** Springer Nature remains neutral with regard to jurisdictional claims in published maps and institutional affiliations.

## Supplementary Material

Supplementary Information

## References

[b1] Yang G. . H_2_S as a physiologic vasorelaxant: hypertension in mice with deletion of cystathionine γ-lyase. Science. 322, 587–590 (2008). 18948540 10.1126/science.1162667 PMC2749494

[b2] Li L. . Hydrogen sulfide is a novel mediator of lipopolysaccharide-induced inflammation in the mouse. FASEB J. 19, 1196–1198 (2005). 15863703 10.1096/fj.04-3583fje

[b3] Kaneko Y., Kimura Y., Kimura H. & Niki I. l-Cysteine inhibits insulin release from the pancreatic β-cell. Diabetes 55, 1391–1397 (2006). 16644696 10.2337/db05-1082

[b4] Shatalin K., Shatalina E., Mironov A. & Nudler E. H_2_S: A universal defense against antibiotics in bacteria. Science 334, 986–990 (2011). 22096201 10.1126/science.1209855

[b5] Mishanina T. V., Libiad M. & Banerjee R. Biogenesis of reactive sulfur species for signaling by hydrogen sulfide oxidation pathways. Nat. Chem. Biol. 11, 457–464 (2015). 26083070 10.1038/nchembio.1834 PMC4818113

[b6] Greiner R. . Polysulfides link H_2_S to protein thiol oxidation. Antioxid. Redox Sign. 19, 1749–1765 (2013). 10.1089/ars.2012.5041 PMC3837443 23646934

[b7] Ida T. . Reactive cysteine persulfides and S-polythiolation regulate oxidative stress and redox signaling. Proc. Natl. Acad. Sci. USA 111, 7606–7611 (2014). 24733942 10.1073/pnas.1321232111 PMC4040604

[b8] Yadav P. K. . Biosynthesis and reactivity of cysteine persulfides in signaling. J. Am. Chem. Soc. 138, 289–299 (2016). 26667407 10.1021/jacs.5b10494 PMC4795164

[b9] Kimura, Y. . Identification of H_2_S_3_ and H_2_S produced by 3-mercaptopyruvate sulfurtransferase in the brain. Sci. Rep. 5, 14774 (2015). 26437775 10.1038/srep14774 PMC4594004

[b10] Abiko Y. . Involvement of reactive persulfides in biological bismethylmercury sulfide formation. Chem. Res. Toxicol. 28, 1301–1306 (2015). 25874357 10.1021/acs.chemrestox.5b00101

[b11] Kimura Y. . Polysulfides are possible H_2_S-derived signaling molecules in rat brain. FASEB J. 27, 2451–2457 (2013). 23413359 10.1096/fj.12-226415

[b12] Paul B. D. & Snyder S. H. H_2_S signalling through protein sulhydration and beyond. Nat. Rev. Mol. Cell Biol. 13, 499–507 (2012). 22781905 10.1038/nrm3391

[b13] Shibuya N. . 3-Mercaptopyruvate sulfurtransferase produces hydrogen sulfide and bound sulfane sulfur in the brain. Antioxid. Redox Signal. 11, 703–714 (2009). 18855522 10.1089/ars.2008.2253

[b14] Whiteman M., Le Trionnaire S., Chopra M., Fox B. & Whatmore J. Emerging role of hydrogen sulfide in health and disease: critical appraisal of biomarkers and pharmacological tools. Clin. Sci. (Lond.) 121, 459–488 (2011). 21843150 10.1042/CS20110267

[b15] Asimakopoulou A. . Selectivity of commonly used pharmacological inhibitors for cystathionine β synthase (CBS) and cystathionine γ lyase (CSE). Br. J. Pharmacol. 169, 922–932 (2013). 23488457 10.1111/bph.12171 PMC3687671

[b16] Porter D. W. & Baskin S. I. The effect of three α-keto acids on 3-mercaptopyruvate sulfurtransferase activity. J. Biochem. Toxicol. 11, 45–50 (1996). 8806051 10.1002/(SICI)1522-7146(1996)11:1<45::AID-JBT6>3.0.CO;2-V

[b17] Wróbel M. & Jurkowska H. Menadione effect on l-cysteine desulfuration in U373 cells. Acta Biochem. Pol. 54, 407–411 (2007). 17520087

[b18] Porter D. W. & Baskin S. I. Specificity studies of 3-mercaptopyruvate sulfurtransferase. J. Biochem. Toxicol. 10, 287–292 (1995). 8934630 10.1002/jbt.2570100602

[b19] Nagahara N., Sawada N. & Nakagawa T. Affinity labeling of a catalytic site, cysteine^247^ in rat mercaptopyruvate sulfurtransferase by chloropyruvate as an analog of a substrate. Biochimie 86, 723–729 (2004). 15556283 10.1016/j.biochi.2004.08.002

[b20] Sasakura K. . Development of a highly selective fluorescent probe for hydrogen sulfide. J. Am. Chem. Soc. 133, 18003–18005 (2011). 21999237 10.1021/ja207851s

[b21] Kawaguchi M. . Fluorescence probe for lysophospholipase C/NPP6 activity and a potent NPP6 inhibitor. J. Am. Chem. Soc. 133, 12021–12030 (2011). 21721554 10.1021/ja201028t

[b22] Zhao Y., Wang H. & Xian M. Cysteine-activated hydrogen hulfide (H_2_S) donors. J. Am. Chem. Soc. 133, 15–17 (2011). 21142018 10.1021/ja1085723 PMC3073703

[b23] Davis B. J. & Erlanson D. A. Learning from our mistakes: The ‘unknown knowns’ in fragment screening. Bioorg. Med. Chem. Lett. 23, 2844–2852 (2013). 23562240 10.1016/j.bmcl.2013.03.028

[b24] Bordo D. & Bork P. The rhodanese/Cdc25 phosphatase superfamily. EMBO rep. 3, 741–746 (2002). 12151332 10.1093/embo-reports/kvf150 PMC1084204

[b25] Sӧrbo B. H. Crystalline rhodanese: II. the enzyme catalyzed reaction. Acta Chem. Scand. 7, 1137–1145 (1953).

[b26] Meyer E. A., Castellano R. K. & Diederich F. Interactions with aromatic rings in chemical and biological recognition. Angew. Chem. Int. Ed. 42, 1210–1250 (2003). 10.1002/anie.200390319 12645054

[b27] *Biochemistry, 7th edition*. Jeremy M Berg, John L Tymoczko, and Lubert Stryer.

[b28] Jarabak R. & Westley J. 3-Mercaptopyruvate sulfurtransferase: rapid equilibrium-ordered mechanism with cyanide as the acceptor substrate. Biochemistry 19, 900–904 (1980). 6928374 10.1021/bi00546a012

[b29] Ploegman J. H. . Nature 273, 124–129 (1978). 643076 10.1038/273124a0

[b30] Yu J. . Structure of AzrA and of AzrC complexed with substrate or inhibit: insight into substrate specificity and catalytic mechanism. Acta Cryst. D70, 553–564 (2014). 10.1107/S1399004713030988 24531489

[b31] Cherney M. M., Zhang Y., Solomonson M., Weiner J. H. & James M. N. G. Crystal structure of sulfide:Quinone oxidoreductase from *Acidithiobacillus ferrooxidans*: insights into sulfidotrophic respiration and detoxification. J. Mol. Biol. 398, 292–305 (2010). 20303979 10.1016/j.jmb.2010.03.018

[b32] Spallarossa A. . The “rhodanese” fold and catalytic mechanism of 3-mercaptopyruvate sulfurtransferases: crystal structure of SseA form *Escherichia coli*. J. Mol. Biol. 335, 583–593 (2004). 14672665 10.1016/j.jmb.2003.10.072

[b33] Alphey M. S., Williams R. A. M., Mottram J. C., Coombs G. H. & Hunter W. N. The crystal structure of *Leishmanis major* 3-mercaptopyruvate sulfurtransferase. J. Biol. Chem. 278, 48219–48227 (2003). 12952945 10.1074/jbc.M307187200

[b34] Bauzá A., Quiñonero D., Deyà P. M. & Frontera A. On the importance of anion-π interactions in the mechanism of sulfide:quinone oxidoreductase. Chem. Asian J. 8, 2708–2713 (2013). 23907989 10.1002/asia.201300786

[b35] Cherney M. M., Zhang Y., James M. N. G. & Weiner J. H. Structure-activity characterization of sulfide:quinone oxidoreductase variants. J. Struct. Biol. 178, 319–328 (2012). 22542586 10.1016/j.jsb.2012.04.007

[b36] Bordo D. . The crystal structure of a sulfurtransferase from *Azotobacter vinelandii* highlights the evolutionary relationship between the rhodanese and phosphatase enzyme families. J. Mol. Biol. 298, 691–704 (2000). 10788330 10.1006/jmbi.2000.3651

[b37] Cianci M., Gliubich F., Zanotti G. & Berni R. Specific interaction of lipoate at the active site of rhodanese. Biochim. Biophys. Acta 1481, 103–108 (2000). 11004580 10.1016/s0167-4838(00)00114-x

[b38] Duan G., Smith V. H. Jr. & Weaver D. F. Characterixation of aromatic-thiol π-type hydrogen bonding and phenylalanine-cysteine side chain interactions through ab initio calculations and protein database analyses. Mol. Phys. 99, 1689–1699 (2001).

[b39] Ringer A. L., Senenko A. & Sherrill C. D. Models of S/π interactions in protein structures: comparison of the H_2_S-benzene complex with PDB data. Prorein Sci. 16, 2216–2223 (2007). 10.1110/ps.073002307 PMC2204139 17766371

[b40] Yadav P. K., Yamada K., Chiku T., Koutmos M. & Banerjee R. Structure and kinetic analysis of H_2_S production by human mercaptopyruvate sulfurtransferase. J. Biol. Chem. 288, 20002–20013 (2013). 23698001 10.1074/jbc.M113.466177 PMC3707699

[b41] Nagahara N. & Nishio T. Role of amino acid residues in the active site of rat liver mercaptopyruvate sulfurtransferase. J. Biol. Chem. 271, 27395–27401 (1996). 8910318 10.1074/jbc.271.44.27395

[b42] Cuevasanta E. . Reaction of hydrogen sulfide with disulfide and sulfenic acid to form the strongly necleophilic persulfide. J. Biol. Chem. 290, 26866–26880 (2015). 26269587 10.1074/jbc.M115.672816 PMC4646399

[b43] Jurkowska H., Placha W., Nagahara N. & Wróbel M. The expression and activity of cystathionine-γ-lyase and 3-mercaptopyruvate sulfurtransferase in human neoplastic. Amino Acids 41, 151–158 (2011). 20446008 10.1007/s00726-010-0606-3

[b44] Frendo J. & Wróbel M. The activity of 3-mercaptopyruvate sulfurtransferase in erythrocytes from patients with polycythemia vera.Acta Biochim. Pol. 44, 771–774 (1997). 9584858

[b45] Nagahara N. . Antioxidant enzyme, 3-mercaptopyruvate sulfurtransferase-knockout mice exhibit increased anxiety-like behaviors: a model for human mercaptolactate-cysteine disulfiduria. Sci. Rep. 3, 01986 (2013). 10.1038/srep01986 PMC3680806 23759691

[b46] Shibuya N. . A novel pathway for the production of hydrogen sulfide from d-cysteine in mammalian cells. Nat. Commun. 4, 1366 (2013). 23340406 10.1038/ncomms2371

[b47] Shibuya N., Mikami Y., Kimura Y., Nagahara N. & Kimura H. Vascular endothelium expresses 3-mercaptopyruvate sulfurtranseferase and produces hydrogen sulfide. J. Biochem. 146, 623–626 (2009). 19605461 10.1093/jb/mvp111

[b48] Otwinowski Z. & Minor W. Processing of X-ray diffraction data collected in oscillation mode. Methods in Enzymol. 276, 307–325 (1997). 10.1016/S0076-6879(97)76066-X 27754618

[b49] Vagin A. & Teplyakov A. *MOLREP*: an Automated Program for Molecular Replacement. J. Appl. Crystallogr. 30, 1022–1025 (1997).

[b50] Emsley P., Lohkamp B., Scott W. G. & Cowtan K. Features and development of *Coot*. Acta Crystallogr. D Biol. Crystallogr. 66, 486–501 (2010). 20383002 10.1107/S0907444910007493 PMC2852313

[b51] Lohkamp B., Emsley P. & Cowtan K. D. Coot News. CCP4 Newsl. 42 (2005).

[b52] Murshudov G. N. . *REFMAC* 5 for the refinement of macromolecular crystal structures. Acta Crystallogr. D Biol. Crystallogr. 67, 355–367 (2011). 21460454 10.1107/S0907444911001314 PMC3069751

[b53] Murshudov G. N., Vagin A. A. & Dodson E. J. Refinement of Macromolecular Structures by the Maximum-Likelihood Method. Acta Crystallogr. D Biol. Crystallogr. 53, 240–255 (1997). 15299926 10.1107/S0907444996012255

[b54] Adams P. D. . *PHENIX*: a comprehensive Python-based system for macromolecular structure solution. Acta Crystallogr. D Biol. Crystallogr. 66, 213–221 (2010). 20124702 10.1107/S0907444909052925 PMC2815670

[b55] ACD/ChemSketch (Freeware) 2012, version 14.01, Advanced Chemistry Development, Inc., Toronto, ON, Canada, www.acdlabs.com (2013).

[b56] Lebedev A. A. . *JLigand*: a graphical tool for the *CCP* 4 template-restraint library. Acta Crystallogr. D Biol. Crystallogr. 68, 431–440 (2012). 22505263 10.1107/S090744491200251X PMC3322602

[b57] Laskowski R. A., MacArthur M. W., Moss D. S. & Thornton J. M. *PROCHECK*: a program to check the stereochemical quality of protein structures. J. Appl. Crystallogr. 26, 283–291 (1993).

[b58] Frisch M. J. . Gaussian 09, Revision D.01. Gaussian, Inc., Wallingford CT, 2013).

[b59] Pople J. A., Head-Gordon M. & Raghavachari K. Quadratic configuration interaction. A general technique for determining electron correlation energies. J. Chem. Phys. 87, 5968–5975 (1987).

[b60] Dunning T. H. Jr. Gaussian basis sets for use in correlated molecular calculations. I. The atoms boron through neon and hydrogen. J. Chem. Phys. 90, 1007–2023 (1989).

[b61] Woon D. E. & Dunning T. H. Jr. Gaussian basis sets for use in correlated molecular calculations. III. The atoms aluminum through argon. J. Chem. Phys. 98, 1358–1371 (1993).

