# Supplementary material for: Discovery and Mechanistic Characterization of Selective Inhibitors of H2S-producing Enzyme: 3-Mercaptopyruvate Sulfurtransferase (3MST) Targeting Active-site Cysteine Persulfide
Source: Sci Rep. 2017 Jan 12;7:40227. doi: 10.1038/srep40227 (PMC5228037; doi:10.1038/srep40227)
Supplement: Supplementary Information [file srep40227-s1.pdf]

Supplementary Information

Discovery and Mechanistic Characterization of Selective Inhibitors of H_2S-producing Enzyme: 3-Mercaptopyruvate Sulfurtransferase (3MST) Targeting Active-site Cysteine Persulfide

Kenjiro Hanaoka[1*], Kiyoshi Sasakura[1], Yusuke Suwanai[1], Sachiko Toma-Fukai[1], Kazuhito Shimamoto[1], Yoko Takano[1], Norihiro Shibuya[2], Takuya Terai[1], Toru Komatsu[1,3], Tasuku Ueno[1], Yuki Ogasawara[4], Yukihiro Tsuchiya[5], Yasuo Watanabe[5], Hideo Kimura[2], Chao Wang[1,6], Masanobu Uchiyama[1,6], Hirotatsu Kojima[7], Takayoshi Okabe[7], Yasuteru Urano[1,8,9], Toshiyuki Shimizu[1] & Tetsuo Nagano[7,*]

[1]Graduate School of Pharmaceutical Sciences, [7]Drug Discovery Initiative and [8]Graduate School of Medicine, The University of Tokyo, 7-3-1 Hongo, Bunkyo-ku, Tokyo 113-0033, Japan. [2]Department of Molecular Pharmacology, National Institute of Neuroscience, National Center of Neurology and Psychiatry, 4-1-1 Ogawa-Higashi, Kodaira, Tokyo 187-8502, Japan. [3]PRESTO, JST, Saitama 332-0012, Japan. [4]Department of Hygienic Chemistry, Meiji Pharmaceutical University, 2-522-1 Noshio, Kiyose, Tokyo 204-8588, Japan. [5]High Technology Research Center, Pharmacology, Showa Pharmaceutical University, Machidashi 194-8543, Tokyo, Japan. [6]Advanced Elements Chemistry Research Team, RIKEN Center for Sustainable Resource Science, and Elements Chemistry Laboratory, RIKEN, 2-1 Hirosawa, Wako-shi, Saitama 351-0198, Japan. [9]AMED CREST (Japan) Agency for Medical Research and Development, Tokyo 100-0004, Japan.

*Correspondence should be addressed to T.N. and K.H. (T.N., (email) tlong@mol.f.u-tokyo.ac.jp; (tel) +81-3-5841-1960; (fax) +81-3-5841-1959) (K.H., (email) khanaoka@mol.f.u-tokyo.ac.jp; (tel) +81-3-5841-4852; (fax) +81-3-5841-4855).

Abbreviations

3MST: 3-mercaptopyruvate sulfurtransferase

3MP: 3-mercaptopyruvate or sodium mercaptopyruvate dihydrate

DMSO: dimethyl sulfoxide

GST: glutathione-S-transferase

HEPES: 2-[4-(2-hydroxyethyl)piperazin-1-yl]ethanesulfonic acid

HRV: human rhinovirus

IPTG: isopropyl β-D-1-thiogalactopyranoside

ITC: isothermal titration calorimetry

OD_{600}: optical density at 600 nm

PEG: polyethylene glycol

PMSF: phenylmethylsulfonyl fluoride

R.m.s.d.: root-mean-square deviation

TCEP: tris(2-carboxyethyl)phosphine

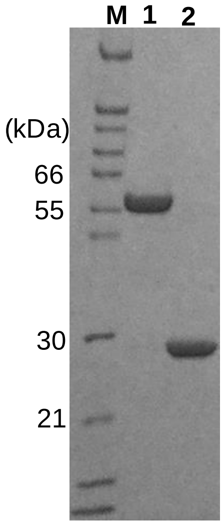

Supplementary Figure S1. SDS-PAGE analysis of GST-3MST and GST. 1: GST-3MST, 2: GST. SDS-PAGE (4-20% polyacrylamide) was performed under denaturing conditions in the presence of β-mercaptoethanol. Proteins were visualized directly by staining with Coomassie brilliant blue.

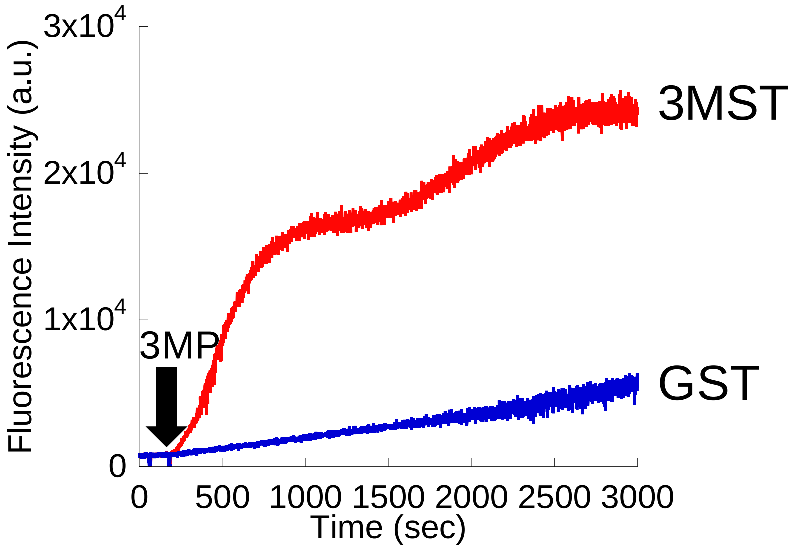

Supplementary Figure S2. The time course of reaction of 1 μM HSip-1 with GST-3MST (30 μg; red) or GST (45 μg; blue) was measured in 30 mM HEPES buffer (pH 7.4) with 100 μM 3MP and 100 μM DTT at 37˚C (total volume: 2005 μL). GST-3MST or GST were added at 60 sec, and 3MP was added at 180 sec. Ex/Em = 491/516 nm. The fluorescence intensity was measured with a Hitachi F4500 (Japan). The time-dependent fluorescence change of the sample containing 100 μM 3MP and 100 μM DTT only without the enzyme, 3MST, was almost the same as the sample containing GST in the above figure.[S1]

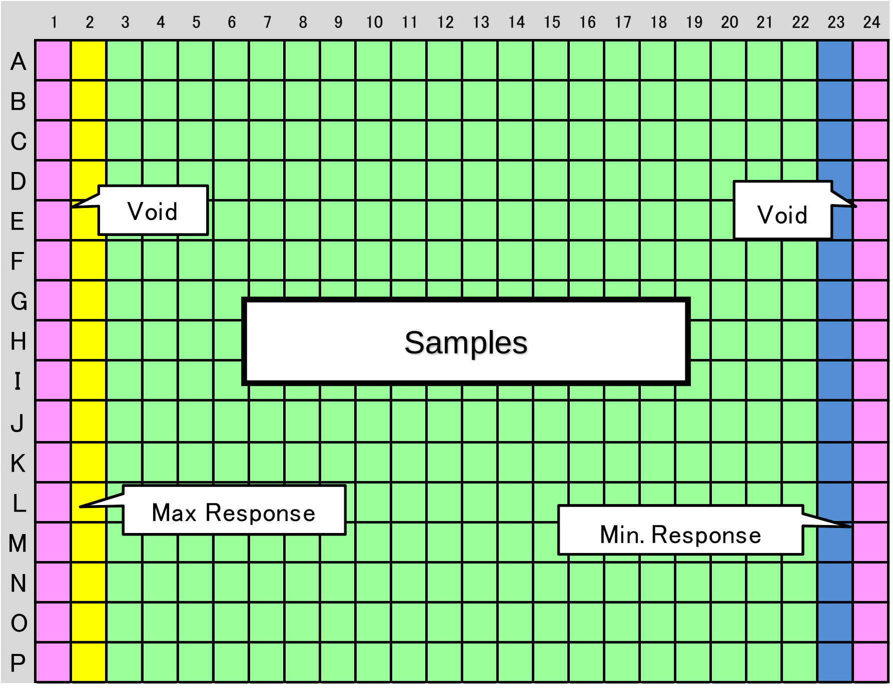

$$\text{InH (\%)} = 100\text{-}100 \times (\text{Value}_{\text{sample}}\text{-Mean}_{\text{Minimum response}})/(\text{Mean}_{\text{Max response}}\text{-Mean}_{\text{Minimum response}})$$

Supplementary Figure S3. Plate layout of high-throughput screening for 3MST inhibitors. For the maximum (Max) response, DMSO was added to the wells as a substitute for the solution of test compound in DMSO. For the minimum (Min) response, GST was added to the wells as a substitute for 3MST.

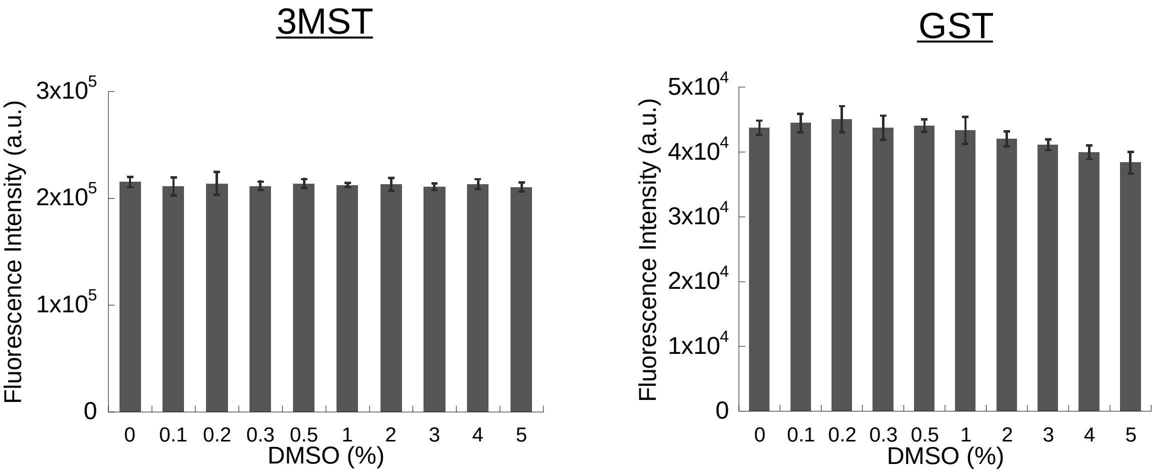

Supplementary Figure S4. The effect of DMSO on enzyme activity. Reactions were performed in 30 mM HEPES buffer (pH 7.4) containing 1 μM HSip-1 at room temperature. The fluorescence intensity of reaction mixtures of 3.8 μg/mL 3MST (left) or 2.2 μg/mL GST (right) with various DMSO concentrations (0, 0.1, 0.2, 0.3, 0.5, 1, 2, 3, 4, 5%) was measured in 30 mM HEPES buffer (pH 7.4) at 3 hr after dispensing 50 μM 3MP and 40 μM DTT in a microplate. The results are mean±S.D. (n = 16). λ_{ex} = 490 nm, λ_{em} = 510 nm.

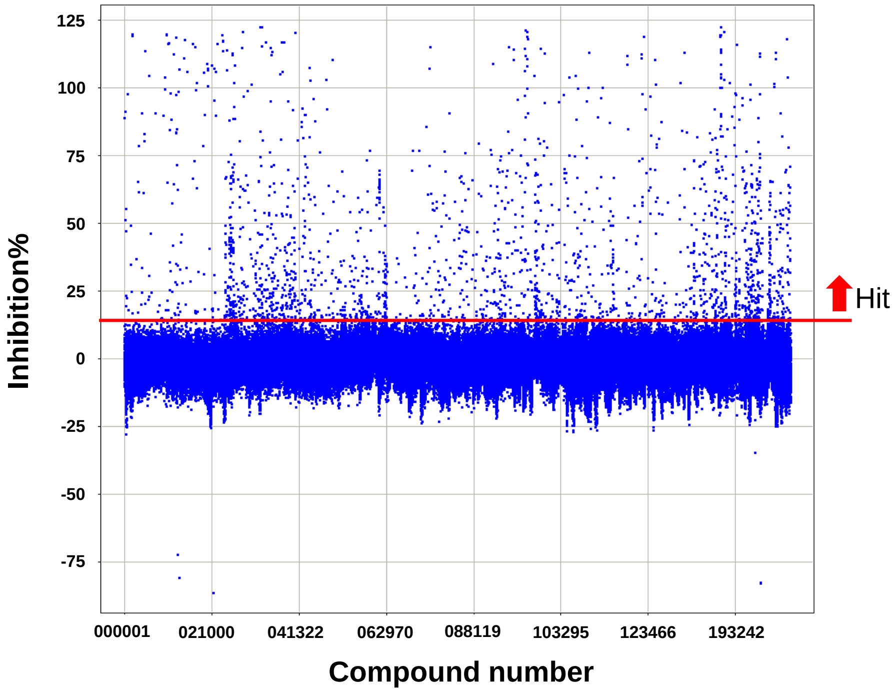

Supplementary Figure S5. Results of primary screening of 3MST inhibitors. Conditions: Mixtures of 1 μM HSip-1, 10 μM test compounds, 3.8 μg/mL 3MST or 2.2 μg/mL GST, 50 μM 3MP, 40 μM DTT, 1% (v/v) DMSO in 30 mM HEPES buffer (pH 7.4) were incubated at room temperature for 3 hr.

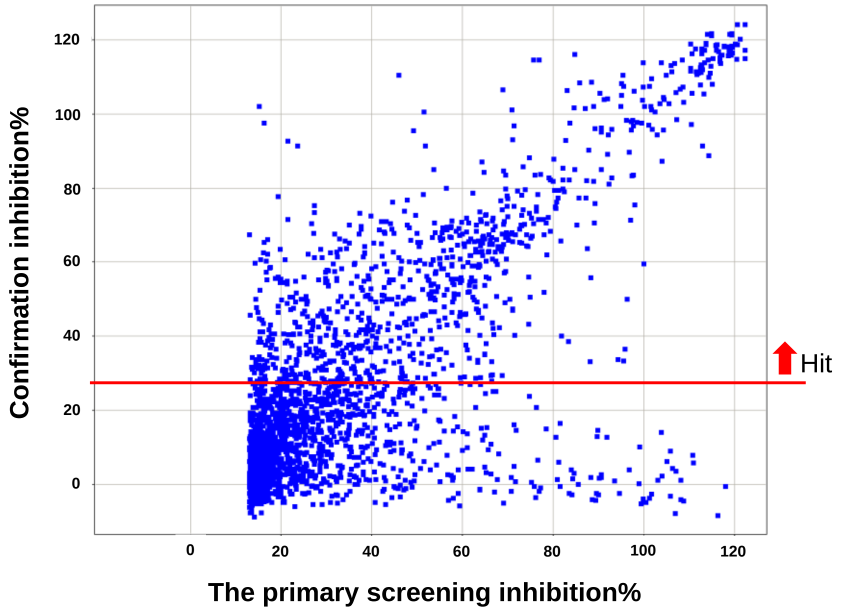

Supplementary Figure S6. Scatter plot of tested compounds (Confirmation test). Each dot indicates inhibition% of 3MST. Horizontal axis and vertical axis indicate the results of the primary screening and the confirmation test, respectively.

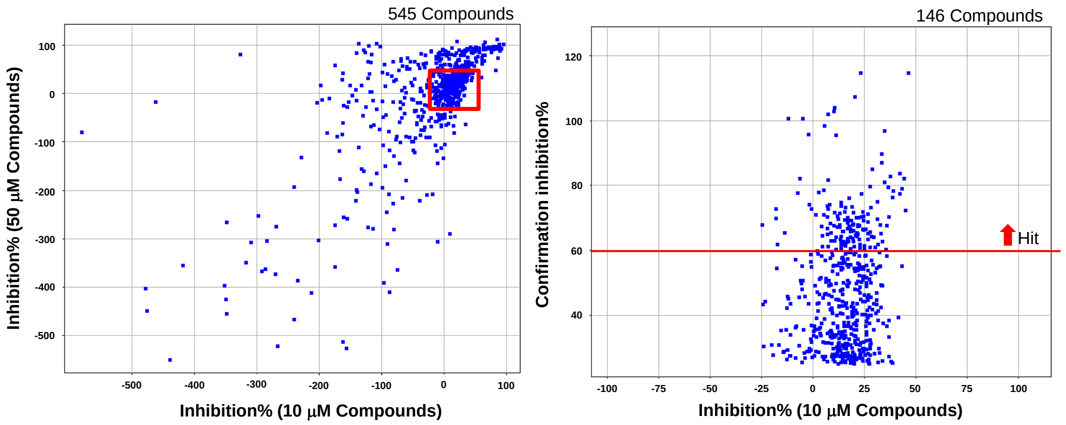

Supplementary Figure S7. Results of screening of thiol-reactive compounds (2nd screening). (left) 545 compounds were selected as thiol-nonreactive compounds. The x-axis and the y-axis show the ability of each compound (10 μM and 50 μM) to inhibit the fluorescence increase of HSip-1 caused by the reaction with 50 μM 3MP and 40 μM DDT, respectively. (right) Selection of compounds with inhibitory activity (InH ≧ 60%) among thiol-nonreactive compounds. 146 compounds were selected. The y-axis shows the inhibitory activity of each compound in the confirmation test. The x-axis shows the ability of each compound (10 μM) to inhibit the fluorescence increase of HSip-1 caused by the reaction with 3MP and DDT.

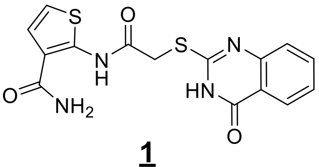

1

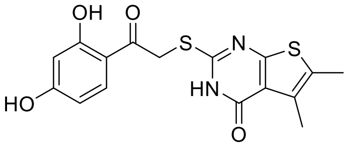

2

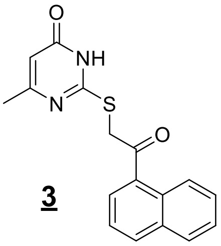

3

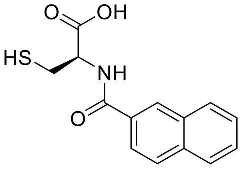

4

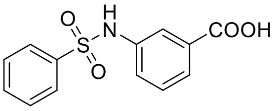

5

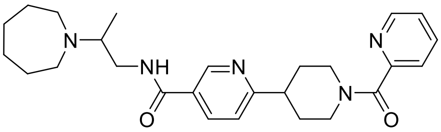

6

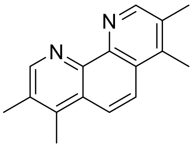

7

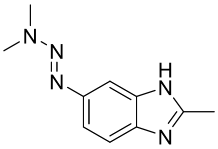

8

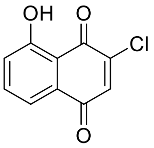

9

Supplementary Figure S8. Chemical structures of hit compounds that showed dose-dependent inhibitory activity against 3MST in the third screening. We calculated IC_{50} values for 3MST inhibition activity of these compounds; however, the IC_{50} value of compound **4** was not determined because its thiol group appeared to be readily oxidized to disulfide.

(a)

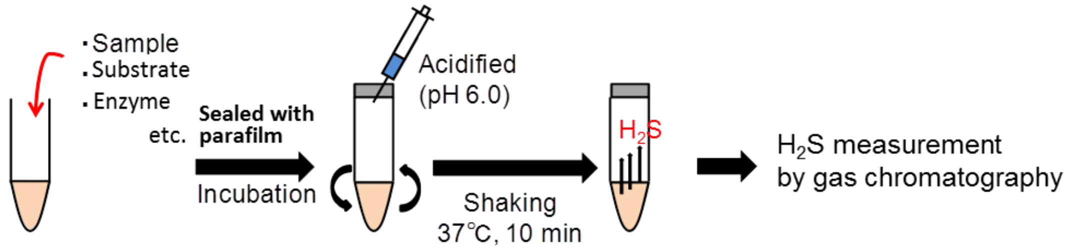

(b)

Compound	Inhibitory activity
**1**	103.7±2.7%
**2**	93.4±0.8%
**3**	90.3±12.0%
**5**	90.9±0.9%

All data are presented as the mean±S.D. (*n* = 2).

Supplementary Figure S9. (a) Schematic illustration of 3MST inhibitor assay by gas chromatography. (b) Inhibitory activity toward recombinant purified m3MST. Conditions: Mixtures of 10 µM test compound, 3.8 µg/mL 3MST or 2.2 µg/mL GST, 50 µM 3MP, 40 µM DTT, and 1% (v/v) DMSO in 30 mM HEPES buffer (pH 7.4) were incubated at room temperature for 3 hr.

Following reagents were prepared.

A. 200 mM KH_2PO_4 buffer, pH 8.6 at 25℃

B. 125 mM sodium thiosulfate ($Na_2S_2O_3$) solution

C. 250 mM potassium cyanide (KCN) solution in reagent A

D. 14% (w/v) nitric acid (HNO_3) solution

E. 410 mM ferric nitrate ($Fe(NO_3)_3$) in reagent D

F. 37% formaldehyde solution

G. 12.5 mM sodium thiosulfate ($Na_2S_2O_3$) solution with 0.025% bovine serum albumin

H. rhodanese solution in reagent G

Procedure:

	**Control**	**TEST**	**Blank**
DMSO or compound	1 μL (DMSO)	1 μL (Compound)	1 μL (DMSO)
Reagent A (Buffer)	23 μL	23 μL	23 μL
Reagent B (Sodium Thiosulfate)	40 μL	40 μL	40 μL
Reagent C (Potassium Cyanide)	20 μL	20 μL	20 μL
Mixed by pipetting and equilibrated to room temperature			
Reagent H (Enzyme Solution)	16 μL	16 μL	-
Mixed by pipetting and incubated at 25℃ for exactly 5 minutes, and then added			
Reagent F (Formaldehyde)	20 μL	20 μL	20 μL
Reagent H (Enzyme Solution)	-	-	16 μL
Mixed by pipetting and then added			
Reagent E (Ferric Nitrate)	100 μL	100 μL	100 μL
Total volume	220 μL	220 μL	220 μL
	↓	↓	↓
Mixed by pipetting, transferred the solution and then added	44 μL	44 μL	44 μL
MilliQ	200 μL	200 μL	200 μL

Mixed by pipetting and recorded the $A_{460\ nm}$ for the Test, Control and Blank using a microplate reader.

Supplementary Figure S10. Assay conditions for measuring rhodanese-inhibitory activity.

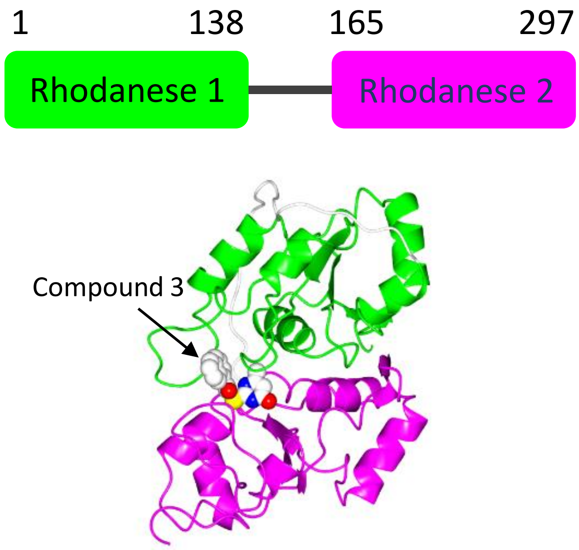

Supplementary Figure S11. Structure of m3MST complexed with compound **3**. m3MST has two rhodanese-like domains (residues 1-138 and 165-297).

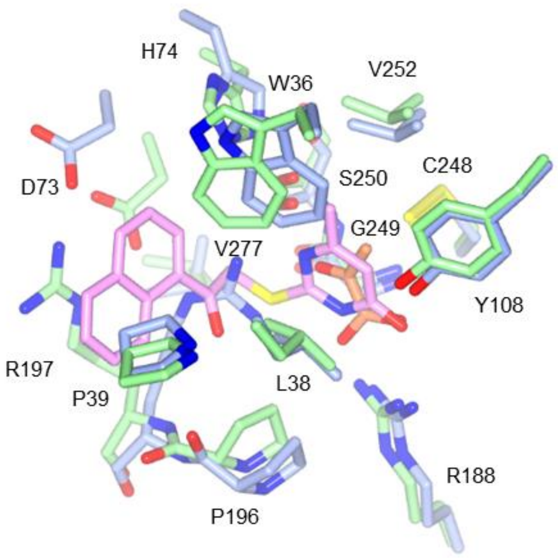

Supplementary Figure S12. Detailed view and superposition of the active site of m3MST-compound **3** complex and h3MST-pyruvate complex (PDB ID: 4JGT). h3MST (purple), m3MST (light green), pyruvate (orange), compound **3** (pink), oxygen atoms (red), nitrogen atoms (blue) and sulfur atoms (yellow) are shown.

Supplementary Table S1. Results of confirmation test, 2nd screening and titration test.

	Confirmation 10 μM	No Enzyme 10 μM	No Enzyme 50 μM	IC_{50} (μM)		Confirmation 10 μM	No Enzyme 10 μM	No Enzyme 50 μM	IC_{50} (μM)
**1**	102.8±0.2%	10.1±6.2%	17.2±4.0%	1.7	**6**	60.5±6.7	18.3±10.6	24.8±4.7	14.2
**2**	81.7±2.5%	7.4±10.6%	-23.6±17.1%	6.3	**7**	114.6±0.5%	23.1±10.6%	23.7±3.6%	2.1
**3**	102.0±2.9%	7.4±5.5%	6.3±4.5%	2.7	**8**	79.6±0.3%	27.8±3.8%	14.2±1.6%	9.4
**4**	98.3±3.8%	5.7±6.2%	-4.1±5.3%	-	**9**	114.5±0.7%	46.3±0.5%	42.4±3.2%	0.68
**5**	95.5±3.4%	11.4±4.9%	10.5±4.5%	5.7					

All data are presented as the mean ± S.D. (n = 4).

Supplementary Table S2. Inhibition (%) of fluorescence increase caused by H_2S in the 3rd screening.

	Compound 10 μM	Compound 30 μM		Compound 10 μM	Compound 30 μM
**1**	0.2±3.5%	1.0±2.4%	**6**	41.2±7.4	99.1±0.9
**2**	6.5±1.8%	19.6±15.3%	**7**	91.4±1.3%	86.6±1.0%
**3**	-3.2±5.0%	-3.5±3.9%	**8**	95.9±0.9%	95.0±3.8%
**4**	2.5±18.0%	-4.9±3.2%	**9**	4.2±3.6%	18.6±14.5%
**5**	2.5±4.5%	-1.2±16.3%			

All data are presented as the mean±S.D. (n = 4). Conditions: Mixtures of 1 μM HSip-1, 10 or 30 μM test compound, 40 μM H_2S donor, 1 mM L-cysteine, and 7.7% DMSO (v/v) were incubated at 37˚C for 60 min. The control with DMSO alone instead of DMSO solution of test compound was taken as 0% inhibition, and the sample without H_2S donor was taken as 100% inhibition.

Supplementary Table S3. Data collection and refinement statistics.

	3MST-compound **1** complex	3MST-compound **3** complex
Data collection		
X-ray source	SPring-8 BL44XU	PF BL-5A
Wavelength (Å)	0.90000	1.00000
Resolution range (Å) (outer shell)	50–1.2 (1.22–1.20)	50–1.7 (1.73–1.70)
Space group	$P2_1$	$P2_1$
Unit-cell parameters		
a (Å)	37.4	37.3
b (Å)	150.4	149.7
c (Å)	54.3	54.0
β (°)	110.1	110.1
No. of observed reflections	629,326	227,437
No. of unique reflections	170,429	59,621
Redundancy (outer shell)	3.7 (3.6)	3.8 (3.8)
Completeness (%) (outer shell)	97.5 (100.0)	98.0 (96.6)
Average I/σ (I) (outer shell)	23.1 (2.3)	14.5 (2.1)
R_{pim} (outer shell)[a]	0.05 (0.45)	0.06 (0.41)
Refinement		
Resolution range (Å)	30.3–1.2	50.0–1.7
R/R_{free} (%)[b]	14.0/17.1	17.5/22.0
Mean B value	13.3	13.9
RMS bond length (Å)	0.015	0.019
RMS bond angle (°)	1.85	1.85
Ramachandran plot (%)[c]		
Favored/allowed/outliers	94.1/5.9/0.0	92.9/7.1/0.0

[a] $R_{\mathrm{pim}} = \sum_{hkl}[1/(N-1)]^{1/2}\sum_i|I_i - \langle I(hkl)\rangle|/\sum_{hkl}\sum_i I_i(hkl)$.

[b] R-factor and $R_{\mathrm{free}} = \sum_{hkl}||F_o|-|F_c||/\sum_{hkl}|F_o|$, where the free reflections (5% of total used) were held aside for R_{free} throughout refinement.

[c] Classifications: favored = residues in most favored regions, allowed = residues in additional allowed and generously allowed regions, outliers = residues in disallowed regions.

Supplementary Table S4. Original calculation data (cartesian coordinates and total energy).

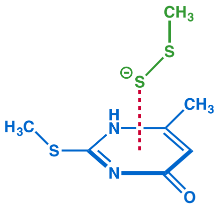

Interraction Model

-1 1

C	-5.33000000	-2.14700000	19.91100000
C	-5.04800000	-1.37800000	18.60500000
S	-4.68100000	-2.37000000	17.18800000
S	-6.39900000	-2.28200000	15.94600000
S	-10.68500000	-5.39000000	16.25000000
C	-10.14800000	-3.96900000	15.42900000
N	-10.27000000	-2.75200000	15.96700000
C	-9.87600000	-1.64800000	15.36900000
O	-10.09200000	-0.52300000	15.89400000
C	-9.29100000	-1.75900000	14.13000000
N	-9.55500000	-4.10500000	14.23000000
C	-9.14400000	-2.99800000	13.59900000
C	-8.42700000	-3.12800000	12.30100000
H	-9.42452838	-5.00807575	13.82081625
H	-7.48957836	-3.61910404	12.45897939
H	-9.02082645	-3.70297740	11.62153614
H	-8.25520240	-2.15536496	11.88945819
H	-4.53633428	-1.97135555	20.60680439
H	-6.25415379	-1.80917733	20.33137557
H	-5.39524403	-3.19421884	19.70129565
H	-5.90599170	-0.77986106	18.37921239
H	-4.21911278	-0.72348997	18.77664671
H	-8.96049327	-0.89094976	13.59881822
C	-9.27453679	-6.33017539	16.79319774
H	-9.58847618	-7.06569291	17.50408173
H	-8.82442730	-6.81579863	15.95267915
H	-8.56284453	-5.67419596	17.24936122

CCSD(T)=-1689.2227905 a.u.

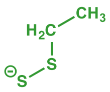

Model of S-S Moiety

-1 1

C	-5.33000000	-2.14700000	19.91100000
C	-5.04800000	-1.37800000	18.60500000
S	-4.68100000	-2.37000000	17.18800000
S	-6.39900000	-2.28200000	15.94600000
H	-4.53633428	-1.97135555	20.60680439
H	-6.25415379	-1.80917733	20.33137557

H	-5.39524403	-3.19421884	19.70129565
H	-5.90599170	-0.77986106	18.37921239
H	-4.21911278	-0.72348997	18.77664671

CCSD(T)=-874.407144 a.u.

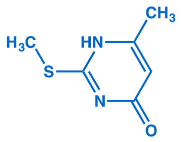

Model of Compound 3

0 1

S	-10.68500000	-5.39000000	16.25000000
C	-10.14800000	-3.96900000	15.42900000
N	-10.27000000	-2.75200000	15.96700000
C	-9.87600000	-1.64800000	15.36900000
O	-10.09200000	-0.52300000	15.89400000
C	-9.29100000	-1.75900000	14.13000000
N	-9.55500000	-4.10500000	14.23000000
C	-9.14400000	-2.99800000	13.59900000
C	-8.42700000	-3.12800000	12.30100000
H	-9.42452838	-5.00807575	13.82081625
H	-7.48957836	-3.61910404	12.45897939
H	-9.02082645	-3.70297740	11.62153614
H	-8.25520240	-2.15536496	11.88945819
H	-8.96049327	-0.89094976	13.59881822
C	-9.27453679	-6.33017539	16.79319774
H	-9.58847618	-7.06569291	17.50408173
H	-8.82442730	-6.81579863	15.95267915
H	-8.56284453	-5.67419596	17.24936122

CCSD(T)=-814.7990645 a.u.

Supplementary Table S5. Donor-acceptor interaction and molecular orbitals.

Interaction	Donor		Acceptor		Δ*E* (kcal/mol)
I	S4	LP1	C8–O9	π*	0.10
II	S4	LP1	C10–O12	π*	0.25
III	S4	LP2	C8–O9	π*	0.22
IV	S4	LP2	C10–O12	π*	0.17

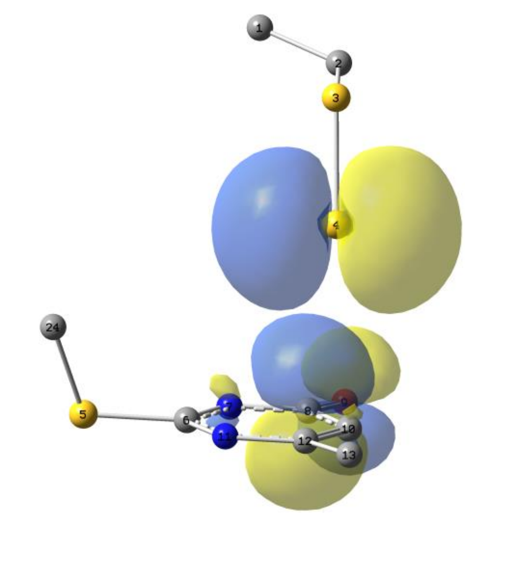

I

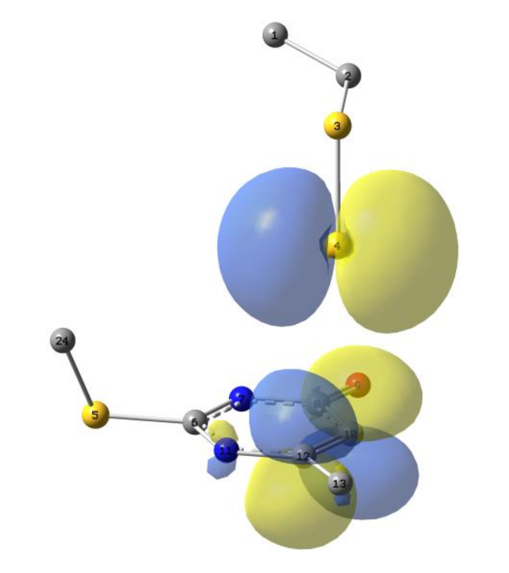

II

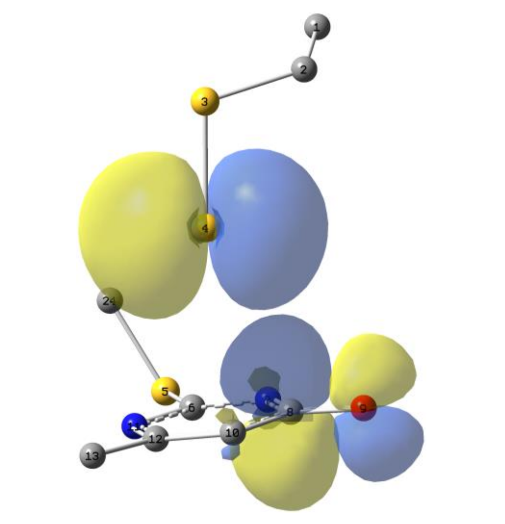

III

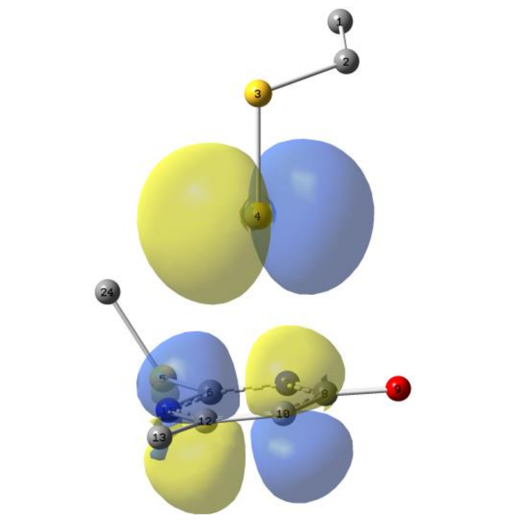

IV

All data were calculated by CCSD(T)/aug-cc-pVDZ.

Supplementary Note 1. Materials and Instruments

General chemicals, except for ITC analysis and X-ray crystallography, were of the best grade available, supplied by Tokyo Chemical Industries, Wako Pure Chemical, Aldrich Chemical Co., Dojindo, and Invitrogen, and were used without further purification. Bovine liver rhodanese (thiosulfate sulfurtransferase, Type II) was purchased from Sigma-Aldrich.

Reagents used in ITC analysis, X-ray crystallography and protein expression (for these analyses) were as follows: Compound **1** was purchased from Enamine, compound **3** was purchased from Otava, and sodium mercaptopyruvate dihydrate was purchased from Sigma-Aldrich. Polyethylene glycol 3,350 Monodisperse (50% w/v solution) (HR2-527), StockOptions HEPES 14 (HR2-902-14) and TCEP hydrochloride were purchased from Hampton Research. Bacto™ Tryptone was purchased from BD. Other reagents were purchased from Nacalai Tesque. All reagents were used as received.

NMR spectra were recorded on a JEOL JNM-LA300 instrument at 300 MHz for ^{1}H NMR and at 75 MHz for ^{13}C NMR, or a JEOL JNM-LA400 instrument at 400 MHz for ^{1}H NMR and at 100 MHz for ^{13}C NMR. δ values are in ppm relative to tetramethylsilane. Mass spectra (MS) were measured with a JEOL JMS-T100LC AccuToF for ESI. The fluorescence spectroscopic studies and the time course measurements of enzymatic reactions were performed on a Hitachi F4500. Reagents for screening were dispensed with Multi Drop Combi (Thermo). Fluorescence assay on 384-well plates was performed using PHERAstar PLUS (BMG LABTECH). A gas chromatograph (ODSA-P2, Alpha M.O.S. Japan) was used for determination of inhibitory activity of hit compounds toward purified 3MST. A gas chromatograph (GC-2014; Shimadzu) and a data processor C-R8A Chromatopac (Shimadzu) were used to examine the selectivity of hit compounds toward purified CSE and CBS. Absorption was measured with a microplate reader (SH-9000; Corona, Electric Co., Ltd., Ibaraki, Japan) for Bradford assay and enzymatic assay of rhodanese.

Supplementary Note 2. Statistics analysis

All statistical analyses of the data were performed using Microsoft Excel 2007 for Windows. The significance of differences between two groups was evaluated with Student's *t* test.

Supplementary Note 3. Details of HTS system for 3MST inhibitors

We performed 3MST inhibitor screening of a chemical library containing 174,118 compounds. All compounds were tested at 10 μM and compounds showing more than 13% inhibitory activity were selected (Primary screening: 2,417 hit compounds) (Supplementary Fig. S3, Supplementary Fig. S5). We also examined the reproducibility (confirmation test) of obtained hit compounds in the primary screening. The same assay of the 2,417 hit compounds was again performed (n = 4) to eliminate false-positives due, for example, to dispensing errors. Also, this time, we selected compounds with more than 25% inhibitory activity (917 hit compounds) (Supplementary Fig. S6).

The second screening, which is a non-enzymatic assay, was performed to eliminate false-positive hit compounds such as naphthoquinone that react with the substrates, e.g., 3MP and DTT. Even in the absence of 3MST, HSip-1 showed a background fluorescence increase upon incubation with 50 μM 3MP and 40 μM DTT. The fluorescence increase in the presence or the absence of 3MP and DTT was regarded as 100% or 0% fluorescence signal, respectively, and we evaluated the ability of each compound (10 μM and 50 μM) to inhibit the fluorescence increase caused by the reaction with 3MP and DTT. In this assay, we selected compounds showing –25% ≤ inhibitory activity at both 10 and 50 μM ≤ 50% (545 compounds). Then, we further selected compounds showing more than 60% inhibitory activity in the previous confirmation test among 545 compounds (146 hit compounds) (Supplementary Fig. S7). These compounds were considered not to react with 3MP and DTT, at least in this concentration range.

In the titration test, we examined the dose-dependence (0.25, 1, 3, 10, 30 μM) of the 3MST-inhibitory activity of each compound (146 hit compounds in the second screening) to determine the half-maximal (50%) inhibitory concentration (IC_{50}). Nine compounds (Supplementary Fig. S8) showed dose-dependent inhibition of 3MST with IC_{50} values in the range of 0.23-14.9 μM (Supplementary Table S1). In the third screening, we excluded false-positive compounds that

directly react with H_2S: For this assay, we synthesized a reported small-molecular H_2S donor[S2] that produces H_2S by reacting with cysteine. Each compound (10 or 30 μM) was dissolved in a solution (30 mM HEPES buffer (pH 7.4)) containing 1 μM HSip-1, 40 μM H_2S donor, 1 mM ʟ-cysteine and 7.7% (v/v) DMSO as a cosolvent, and the solution was incubated at 37˚C for 60 min. Then, the fluorescence intensities were measured to evaluate the amount of H_2S in the solution (Supplementary Table S2). Based on the results of the titration test and the third screening, we concluded that compound **9** showed weak reactivity with thiol compounds such as 3MP and DTT, while compounds **6-8** appeared to react with H_2S. Compound **4** was excluded because it has a thiol group and appeared to be readily oxidized to the disulfide. On the other hand, compounds **1-3**, **5** showed >80% inhibition of 3MST enzymatic activity at 10 μM, and their IC_{50} values were in the range of 2-7 μM (Supplementary Table S1). Interestingly, **1-3** have similar structural scaffolds, i.e., an aromatic ring-carbonyl-*S*-pyrimidone structure (Supplementary Fig. S8). This structure may be important for inhibitory activity towards 3MST. Based on its structure, compound **9** seems to be one of the pan-assay interference compounds (PAINS), which show activity across a range of assay platforms and against a range of proteins[S3], so we excluded this compound from the hit compounds. We then confirmed the inhibitory activity of the remaining 4 hit compounds by directly monitoring H_2S production by gas chromatography, because we had detected H_2S production only with the fluorescent probe up to this point. All 4 compounds showed >80% inhibition at 10 μM, in accordance with the results obtained with the fluorescent probe (Supplementary Fig. S9).

Supplementary References:

S1. Sasakura, K., Hanaoka, K., Shibuya, N., Mikami, Y., Kimura, Y., Komatsu, T., Ueno, T., Terai, T., Kimura, H. & Nagano, T. Development of a highly selective fluorescent probe for hydrogen sulfide. *J. Am. Chem. Soc.* **133**, 18003–18005 (2011).

S2. Zhao, Y., Wang, H. & Xian, M. Cysteine-activated hydrogen hulfide (H_2S) donors. *J. Am. Chem. Soc.* **133**, 15–17 (2011).

S3. Davis, B. J. & Erlanson, D. A. Learning from our mistakes: The 'unknown knowns' in fragment screening. *Bioorg. Med. Chem. Lett.* **23**, 2844–2852 (2013).
